# Terminal dysprosium and holmium organoimides†

**DOI:** 10.1039/d3sc06584g

**Published:** 2024-01-22

**Authors:** Theresa E. Rieser, Dorothea Schädle, Cäcilia Maichle-Mössmer, Reiner Anwander

**Affiliations:** a Institut für Anorganische Chemie, Eberhard Karls Universität Tübingen Auf der Morgenstelle 18 72076 Tübingen Germany reiner.anwander@uni-tuebingen.de

## Abstract

Terminal rare-earth-metal imide complexes Tp^*t*Bu,Me^Ln(NC_6_H_3_iPr_2_-2,6)(dmap) of the mid-late rare-earth elements dysprosium and holmium were synthesized *via* double methane elimination of Lewis acid stabilized dialkyl precursors Tp^*t*Bu,Me^LnMe(GaMe_4_) with primary aniline derivative H_2_NC_6_H_3_iPr_2_-2,6 (H_2_NAr^iPr^). Exploiting the weaker Ln–CH_3_⋯[GaMe_3_] interaction compared to the aluminium congener, addition of the aniline derivative leads to the mixed methyl/anilido species Tp^*t*Bu,Me^LnMe(HNAr^iPr^) which readily eliminate methane after being exposed to the Lewis base DMAP (=*N*,*N*-dimethyl-4-aminopyridine). Under the same conditions, [AlMe_3_]-stabilized dimethyl rare-earth-metal complexes transform immediately to Lewis acid bridged imides Tp^*t*Bu,Me^Ln(μ_2_-NC_6_H_3_Me_2_-2,6)(μ_2_-Me)AlMe_2_ (Ln = Dy, Ho). DMAP/THF donor exchange is accomplished by treatment of Tp^*t*Bu,Me^Ln(NC_6_H_3_iPr_2_-2,6)(dmap) with 9-BBN in THF while the terminal imides readily insert carbon dioxide to afford carbamate complexes.

## Abstract 2

Tp^*t*Bu,Me^Ln(=NC_6_H_3_iPr_2_-2,6)(dmap) (Ln = Dy, Ho) display the first terminal imides of open-shell mid-sized lanthanides, exhibiting reactivity similar to Chen's seminal scandium terminal imide.

## Graphical abstract

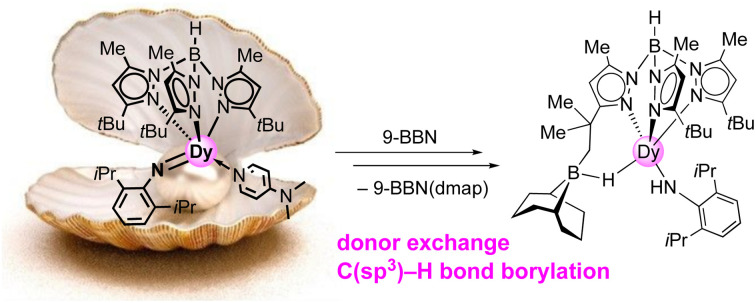

## Introduction

**Scheme 1:**
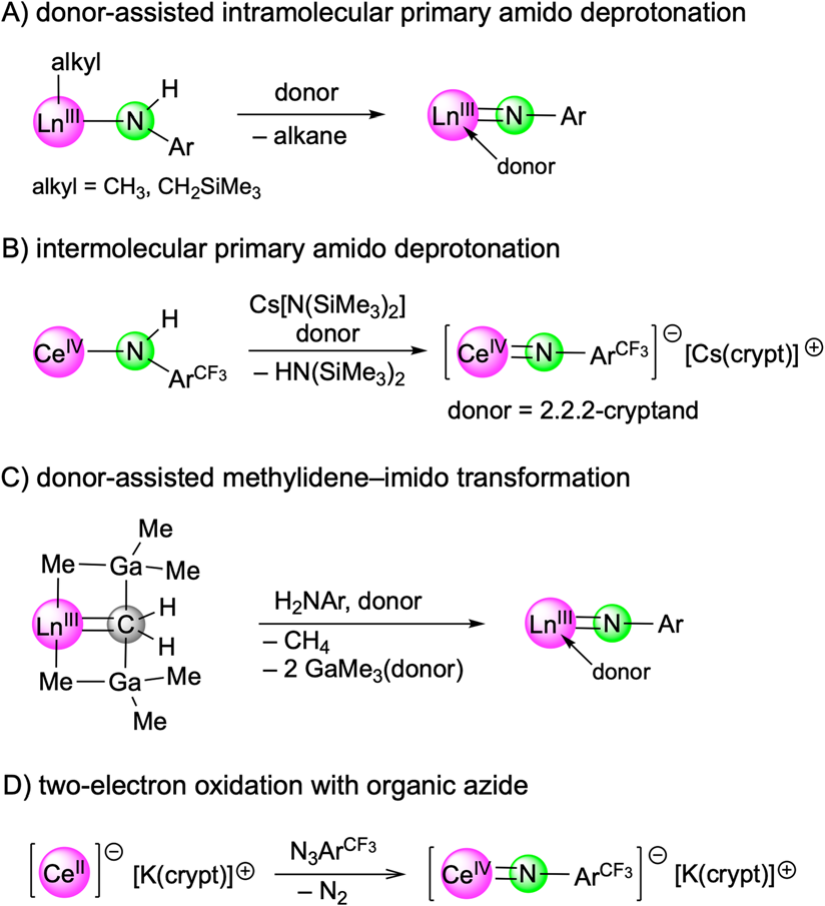
Synthesis strategies for terminal rare-earth-metal imides.

Recent years have witnessed major progress in the field of rare-earth-metal (Ln) complexes with multiply bonded (dianionic) main-group ligands, most notably (organo)imide chemistry.^1–3^ Upon closer inspection this is not all that surprising, since [Ln=NR] moieties display a favourable Pearson hard/hard match and enhanced steric/electronic variability through the imido substituent R,^4^ compared to other fragments such as [Ln=O],^5^ [Ln=PR],^6^ or [Ln=CR_2_].^7^ Of particular interest have been the synthesis of terminal rare-earth-metal imides^8–15^ and a fundamental understanding of the Ln–imido bonding as well as reactivity,^16^ and in particular small-molecule-activation scenarios.^17^ Closely based on Chen's synthesis protocol of the first terminal scandium imide complex L^1^Sc(NAr^iPr^)(dmap) (Ar^iPr^ = C_6_H_3_iPr_2_-2,6; L^1^ = [Ar^iPr^NC(Me)CHC(Me)N(CH_2_)_2_NMe_2_]),^8^ a donor-promoted intramolecular methane/tetramethylsilane elimination from mixed methyl(neosilyl)/primary amido precursors emerged as the most efficient approach for accessing terminal Ln(iii) imides (Scheme 1/A).^8–13^ Meanwhile, another three strategies have been successfully pursued for the early larger rare-earth metals. While anionic terminal ceric imides could be obtained by the deprotonation of a neutral primary amide complex with alkali-metal silylamides (Scheme 1/B),^14^ a [Ln=CH_2_(GaMe_3_)_2_] → [Ln=NR(thf)_2_] transformation proved feasible for generating the open-shell terminal imides Tp^*t*Bu,Me^Ln(NAr^iPr^)(thf)_2_ (Ln = Ce, Nd, Sm) (Scheme 1/C).^15^ More recently, an anionic cerium(iv) terminal imide was accessed by a two-electron oxidation of a “Ce(ii)” complex supported by a tripodal tris(amido)arene ligand using azide N_3_Ar^CF_3_^ (Ar^CF_3_^ = C_6_H_3_(CF_3_)_2_-3,5) (Scheme 1/D).^18^ Less surprising, terminal imides of the extremely large divalent rare-earth-metal centres have remained elusive.^19^

Common features of all terminal rare-earth-metal imides, reported so far, are that the imido ligand is derived from a substituted aniline, H_2_NAr^R^ (R = iPr, Me, CF_3_), and an indispensable kinetic stabilization by use of sterically demanding ancillary co-ligands. Aliphatic amines, benzylic amines, and silylamines engage in imido ligand formation as well but have been detected only as metal-bridging and Lewis acid stabilized versions.^3^ Successfully applied ancillaries include β-diketiminato (nacnac),^8,9,11^ phosphazene,^10^ TriNox,^14^ and multidentate pyrazolato ligands.^12,13,15^ We found that especially the bulky monoanionic scorpionato ligand hydrotris(3-*tert*-butyl-5-methylpyrazolyl)borato (Tp^*t*Bu,Me^) provides a useful scaffold for stabilizing terminal imides as well as phosphinidenes.^6*c*,12,15^ However, like for all terminal Ln(III) imides, stabilization of the highly polarized Ln=N bond, which predominantly consists of non-directional ionic interactions, is still challenging as it readily reacts with solvent molecules or the ancillary ligand of the aspired complexes.^2,3^ DFT calculations performed on the yttrium compound Tp^*t*Bu,Me^Y(NC_6_H_3_Me_2_-2,6)(dmap) confirmed a large ionic character of the Y–N(imido) bond but also a significant covalent bonding pattern with one σ-type and two π-type interactions.^6*c*^

Given the feasibility of terminal imides of the early open-shell cations Ce(iii) (f^1^), Nd(iii) (f^3^), and Sm(iii) (f^5^),^15^ we herein envisaged the synthesis of those of the mid-late open-shell cations Dy(iii) (f^9^) and Ho(iii) (f^10^). These metal centres exhibit ionic radii similar to Y(iii) but would (if at all) contribute to a distinct covalent bonding. On the other hand, their much higher molar mass might promote the crystallization behaviour and hence, the single-crystal X-ray structure diffraction (SCXRD) analysis of the targeted complexes. We also examined the reactivity of the first terminal, trivalent dysprosium and holmium imide complexes.

## Results and discussion

### Selection of precursors

**Scheme 2:**
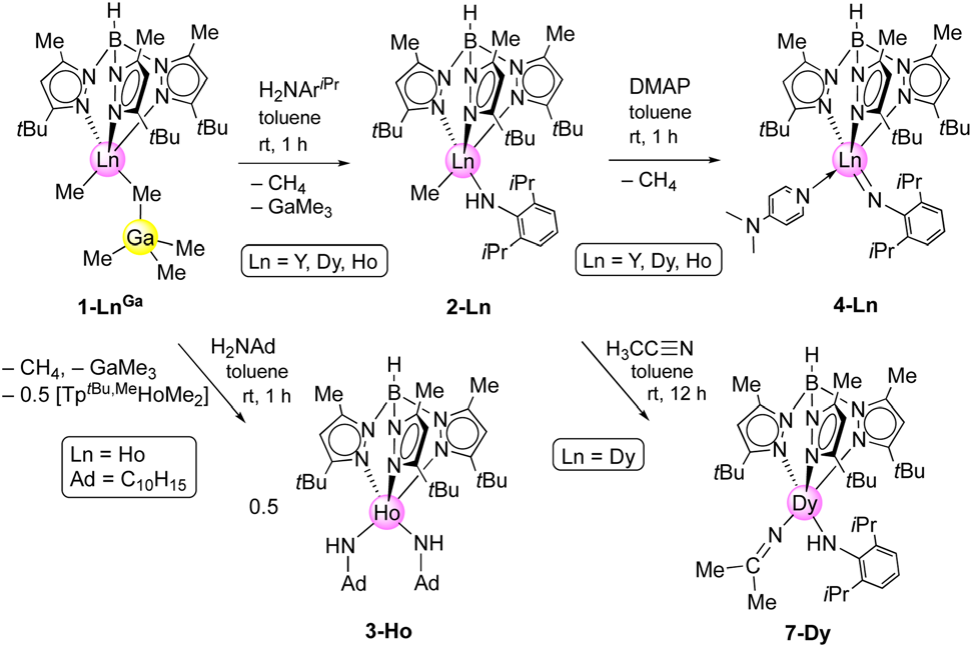
Formation of Tp^*t*Bu,Me^Ln(NAr^iPr^)(dmap) (4-Ln: Ln = Y, Dy, Ho) *via* reaction of Tp^*t*Bu,Me^LnMe(GaMe_4_)] (1-Ln^Ga^) with primary aniline H_2_NAr^iPr^ to afford mixed methyl/amido complexes Tp^*t*Bu,Me^LnMe(HNAr^iPr^) (2-Ln), and subsequent addition of DMAP. Use of 1-adamantylamine led to bis(amido) complex [Tp^*t*Bu,Me^Ho(HNAd)_2_] (3-Ho). Nucleophilic attack of acetonitrile by Dy–CH_3_ affords 7-Dy.

Our original approach toward the terminal yttrium imide Tp^*t*Bu,Me^Y(NC_6_H_3_Me_2_-2,6)(dmap) involved the mixed methyl/tetramethylgallato complex Tp^*t*Bu,Me^YMe(GaMe_4_) as a suitable precursor.^12^ There, the ease of GaMe_3_ displacement proved to be crucial for the successful synthesis. Consequently, we chose the trimethylgallium-stabilized dialkyl complexes Tp^*t*Bu,Me^LnMe(GaMe_4_) (1-Ln^Ga^; Ln = Y,^12^ Dy,^6*c*^ Ho) as precursors for the present study. Like their aluminium congeners,^20^ complexes 1-Ln^Ga^ are available in moderate yield *via* protonolysis of the homoleptic gallates Ln(GaMe_4_)_3_ (ref. 21) with H[Tp^*t*Bu,Me^] (ref. 22) and precipitation from toluene or *n*-hexane solution (Scheme 2; for detailed metrics of Ln(GaMe_4_)_3_ (Dy, Ho) and 1-Ho^Ga^; see the ESI, Fig. S1–S3†). The solid-state structure of the bimetallic compounds Tp^*t*Bu,Me^LnMe(μ_2_-MeEMe_3_) (1-Ln^E^, E = Al, Ga), depicting one terminal methyl group and an almost linear Ln–Me–E linkage, is not reflected in the solution NMR spectra, which reveal highly fluxional methyl groups at ambient temperature, an even higher mobility in case of the gallium derivatives. The isostructural 1-Ln^Ga^ show Ln–C(Me) (Dy: 2.389(3) Å,^6*c*^ Y: 2.385(3) Å,^12^ Ho: 2.356(5) Å) and Ln–C(Me_Ga_) distances (Dy: 2.736(2) Å,^6*c*^ Y: 2.688(2) Å,^12^ Ho: 2.652(4) Å) in accordance with the distinct Ln(iii) radii.^23^ Unexpectedly, the solid-state structures of the formally five-coordinate 1-Ln^Ga^ of the similar sized yttrium and holmium differ in the hapticity of the Tp^*t*Bu,Me^ ligand and in the Ln1–C26–Ga1 angle, which is more linear for the holmium derivative (174.9(2) *vs.* 163.3(1)°). Packing effects of co-crystallizing toluene in 1-Ho^Ga^ have presumably a major impact on the coordination of the GaMe_4_ moiety and might also cause the bending of one pyrazolyl moiety toward the rare-earth metal centre. The pyrazolyl nitrogen atoms exhibit Ho–N interatomic distances ranging from 2.334(3) to 2.376(3) Å with an additional close contact to the tilted pyrazolato ligand (Ho⋯N5, 2.859(3) Å, see Fig. S3†). The proposed mechanism for the formation of 1-Ln^Ga^ includes the preformation of [Tp^*t*Bu,Me^Ln(GaMe_4_)_2_] under release of methane and trimethylgallium, and the elimination of a second molecule GaMe_3_ sterically induced by the bulky Tp^*t*Bu,Me^ ligand.

The primary aniline H_2_NAr^iPr^ (Ar^iPr^ = C_6_H_3_iPr_2_-2,6) has proven a privileged imido ligand precursor in rare-earth-metal chemistry.^1–3^ The molecule not only features the right balance of adequately acidic protons, but also a sufficient steric protection with bulky substituents in the positions 2 and 6 at the aryl group. The reaction of bis(alkyl) 1-Ln^Ga^ with H_2_NAr^iPr^ to form the mixed methyl/primary amido complexes Tp^*t*Bu,Me^LnMe(HNAr^iPr^) 2-Ln (Ln = Y, Dy, Ho) is clearly visible by the elimination of methane and displacement of trimethylgallium (Scheme 2).

Complexes 2-Dy and 2-Ho are isostructural but crystallize in different space groups (2-Dy: *P*2_1_/*c*; 2-Ho: *P*1̄; for the crystal structures and detailed metrics, see the ESI, Fig. S4/S5†). Both compounds are insoluble in *n*-hexane, but dissolve in aromatic solvents such as toluene. The ancillary ligand coordinates in a κ^3^ fashion (*N*, *N*′, *N*′′) with considerably varying Ln–N_pz_ distances (2-Dy: 2.3752(17)–2.5163(18) Å; 2-Ho: 2.369(2)–2.536(2) Å). The Ln–N_amido_ (2-Dy: 2.212(2) Å; 2-Ho: 2.222(2) Å) and the Ln–CH_3_ (2-Dy: 2.436(2) Å; 2-Ho: 2.427(3) Å) interatomic distances lie in the range of the similar mixed methyl/amido complexes Tp^*t*Bu,Me^LuMe(HNAr^R^) (Ar^R^ = Ar^Me_2_^ = C_6_H_3_Me_2_-2,6: Lu–N_amido_ 2.189(2) Å, Lu–CH_3_ 2.369(2) Å; Ar^R^ = Ar^CF_3_^ = C_6_H_3_(CF_3_)_2_-3,5: Lu–N_amido_ 2.215(1) Å, Lu–CH_3_ 2.360(1) Å).^12^ The Ln–N_amido_–C_ipso_ angle spans a wide range from 142.5(1)° (Ln = Lu, Ar^R^ = Ar^CF3^),^12^ 153.8(1)° (Ln = Lu, Ar^R^ = Ar^Me2^)^12^ over 155.84(16)° (Ln = Dy, Ar^R^ = Ar^iPr^) to 160.28(19)° (Ln = Ho, Ar^R^ = Ar^iPr^), dependent on the Ln(III) centre and the substitution pattern of the amido ligand. As the paramagnetic nature of dysprosium and holmium impedes conclusive interpretations of their NMR spectra, the yttrium congener 2-Y was accessed from 1-Y^Ga^ and H_2_NAr^iPr^. The ^1^H NMR spectrum of 2-Y shows a sharp singlet at 0.46 ppm for the methyl group and a broader singlet at 4.87 ppm for the proton of the amido ligand.

**Fig. 1:**
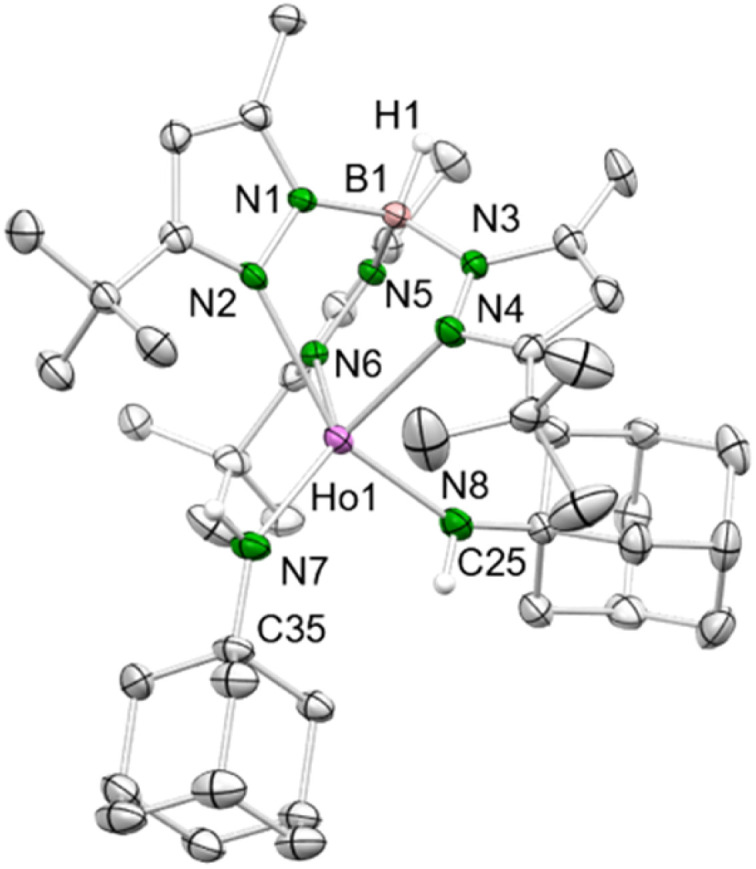
Crystal structure of 3-Ho. All atoms are represented by atomic displacement ellipsoids set at 50% probability. Solvent molecules and hydrogen atoms except for those of B–H and N–H are omitted for clarity. Selected interatomic distances (Å) and angles (°): Ho1–N7 2.172(3), Ho1–N8 2.170(3); Ho1–N7–C35 144.0(3), Ho1–N8–C25 148.5(3) (for further metrics, see ESI†).

Noteworthy, the reaction of 1-Ho^Ga^ with 1-adamantylamine in a 0.9 : 1 ratio gave the bis(amido) holmium complex Tp^*t*Bu,Me^Ho(HNAd)_2_ (3-Ho, Ad = adamantyl), even though the primary amine was added in deficit. Apparently, the single deprotonation is favored over the second deprotonation, as the less Brønsted acidic second proton is less prone to be abstracted than the first proton of a second primary aniline. According to the present synthesis protocol, so far only sufficiently acidic aniline derivatives lead to the successful isolation of terminal rare-earth-metal imides, as other, less bulky and less electronically advantageous amines only form bis(amido) complexes,^3,6*c*^ or in case 1-Ln^Al^ are employed result in trimethylaluminium-stabilized imide species.^20*a*^ Like its precursors, complex 3-Ho is insoluble in aliphatic solvents, but readily dissolves in toluene and THF. The crystal structure of 3-Ho (triclinic *P*1̄ space group) shows the expected κ^3^ fashion (*N*, *N*′, *N*′′) of the ancillary ligand with Ho–N_pz_ distances in the range of 2.393(3)–2.610(3) Å (Fig. 1).

The Ln–N_amido_ distances (*ø* 2.171 Å) are longer compared to the Ln–N_imido_ interactions in Tp^*t*Bu,Me^Ho(μ_2_-NAd)AlMe_3_ (2.087(2) Å),^20*a*^ but slightly shorter compared to the mixed methyl/amido complexes 2-Ln (Ln = Dy, Ho) described beforehand. This reflects a better electron donation of two nitrogen atoms to the metal centre, despite increased steric bulk. However, the angles Ho1–N7–C35 (144.0(3)°) and Ho1–N8–C25 (148.5(3)°) are significantly more bent compared to the methyl/anilido complex 2-Ho (160.28(19)°).

### Ln(iii) imide synthesis

Treatment of the mixed methyl/anilido complexes 2-Ln with the Lewis base DMAP led to the isolation of the targeted terminal lanthanide imide complexes Tp^*t*Bu,Me^Ln(NAr^iPr^)(dmap) (4-Ln: Ln = Y, Dy, Ho). DMAP was used previously for the synthesis of terminal rare-earth-metal imide complexes,^8,10–12^ exploiting its strong donor capacity (*versus e.g.*, THF) to induce the elimination of methane *via* inner-sphere deprotonation of the amido species. We assume, that the preceding coordination of DMAP to the metal centre affects the geometry of its coordination sphere in a way that the methyl group and the amido proton come into close proximity and finally evolve methane, being a very potent leaving group. The ^1^H NMR spectrum of 4-Y evidences the deprotonation of the amido functionality *via* the methyl ligand since both the N–H and methyl signal disappeared (see Fig. S21†). Complexes 4-Ln are insoluble in non-polar solvents (*e.g. n*-hexane), but easily dissolve in aromatic or polar solvents like toluene and THF.

**Fig. 2:**
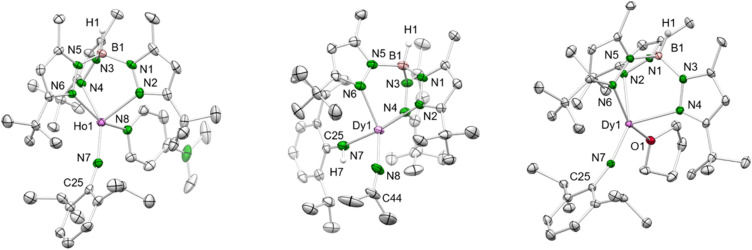
Left: Crystal structure of 4-Ho. All atoms are represented by atomic displacement ellipsoids set at 50% probability. Solvent molecules, and hydrogen atoms except for that of B–H are omitted for clarity. For selected interatomic distances and angles, see Table 1 and ESI.† Middle: Crystal structure of 7-Dy. All atoms are represented by atomic displacement ellipsoids set at 50% probability. Solvent molecules and hydrogen atoms except for those of B–H and N–H are omitted for clarity. Selected interatomic distances (Å) and angles (°): Dy1–N7 2.219(3), Dy1–N8 2.148(3); Dy1–N7–C25 156.3(3), Dy1–N8–C44 165.3(3) (for further metrics, see ESI†). Right: Crystal structure of 8-Dy. All atoms are represented by atomic displacement ellipsoids set at 50% probability. Only one molecule of the asymmetric unit is shown. Solvent molecules, and hydrogen atoms except for that of B–H are omitted for clarity. For selected interatomic distances and angles, see Table 1 and ESI.†

While the yttrium complex 4-Y could not be obtained in very pure form and gave only poor crystal quality (connectivity structure only, see ESI Fig. S7†), the dysprosium and holmium congeners displayed good crystallization and diffraction behaviours (isotypic, monoclinic space group *C*2/*c*, Fig. 2 and S8/9†). The metal centres are pentacoordinate with the ancillary Tp^*t*Bu,Me^ ligand coordinating in the familiar κ^3^ fashion (*N*, *N*′, *N*′′). The Ln–N_pz_ distances typical of scorpinate ligands show two shorter bonds (4-Dy: 2.450(3)/2.452(3) Å, 4-Ho: 2.426(4)/2.436(3) Å) and one longer (4-Dy: 2.517(3) Å, 4-Ho: 2.492(4) Å). The Ln–N_imido_ distances are in line with those of other terminal imides, considering the changes in the ionic radii (Table 1). Except for the anionic ceric complex [(TriNOx)Ce(NAr^CF_3_^)[Cs(2.2.2-cryptand)] (157.3(4)°),^14*a*^ the Ln–N_imido_–C_ipso_ angles of all trivalent terminal imides are larger than 165° and almost linear (4-Dy: 166.0(2)°; 4-Ho: 166.7(3)°; Table 1). Exchange of the donor ligand dmap for thf seems to entail a shortening of the Ln–N_imido_ bond but doesn't appear to affect the Ln–N_imido_–C_ipso_ angle.

**Table 1:** Selected metrical parameters of terminal rare-earth-metal imides

Compound	Ln=N_imido_/Å	Ln–do/Å	Ln–N_imido_–C_ipso_/deg	IR^d^/Å (ref. 23)	CN	Ref.
Tp^*t*Bu,Me^Lu(NAr^CF_3_^)(dmap)	1.993(5)	2.377(5)	175.8(5)	0.861	5	12
Tp^*t*Bu,Me^Y(NAr^Me_2_^)(dmap)	2.024(4)	2.426(4)	173.6(4)	0.900	5	12
Tp^*t*Bu,Me^Ho(NAr^iPr^)(dmap) (4-Ho)	2.012(4)	2.429(4)	166.7(3)	0.901	5	This work
Tp^*t*Bu,Me^Dy(NAr^iPr^)(dmap) (4-Dy)	2.017(3)	2.450(3)	166.0(2)	0.912	5	This work
Tp^*t*Bu,Me^Dy(NAr^iPr^)(thf) (8-Dy)^a^	2.008(3)/2.004(4)	2.397(3)/2.392(3)	166.9(3)/165.3(3)	0.912	5	This work
Tp^*t*Bu,Me^Sm(NAr^iPr^)(thf)_2_	2.067(5)	2.527(5)/2.560(4)	169.3(5)	0.958	6	15
Tp^*t*Bu,Me^Nd(NAr^iPr^)(thf)_2_	2.076(4)	2.557(4)/2.594(3)	169.2(4)	0.983	6	15
Tp^*t*Bu,Me^Nd(NAr^iPr^)(thf)^a^	2.036(7)/2.047(7)	2.517(6)/2.511(6)	165.8(4)/163.2(6)	0.983	5	15
Tp^*t*Bu,Me^Ce(NAr^iPr^)(thf)_2_	2.101(5)	2.599(3)/2.628(3)	171.3(3)	1.01	6	15
(nacnac^R1^)Sc(NAr^iPr^)(dmap)^b^	1.881(8)	2.271(5)	169.6(5)	0.745	5	8
(nacnac^R1^)Sc(NAr^iPr^)(thf)^b^	1.852(4)	2.251(3)	168.6(3)	0.745	5	11
(nacnac^R2^)Sc(NAr^iPr^)(dmap)^c^	1.8591(18)	2.369(2)	167.90(17)	0.745	5	9
[(PhN = Ph_2_P)_2_N]Sc(NAr^iPr^)(dmap)_2_	1.853(3)	2.379(3)/2.326(3)	168.8(3)	0.745	6	10
(BPz_2_Py_3_)Sc(NAr^iPr^)	1.877(3)	—	173.1(3)	0.745	6	13
[(TriNOx)Ce(NAr^CF_3_^)][Cs(2.2.2-cryptand)]	2.077(3)	—	157.3(4)	0.87	8	14*a*
[(^Ad^TPBN_3_)Ce(NAr^CF_3_^)][K(2.2.2-cryptand)]	2.0742(4)	—	176.3(4)	<0.87	4	18

a Two molecules in the asymmetric unit.

b nacnac^R1^ = [Ar^iPr^NC(Me)CHC(Me)N(CH_2_)_2_NMe_2_].

c nacnac^R2^ = [Ar^iPr^NC(Me)CHC(Me)N(CH_2_)_2_N(CH_2_)_2_NMe_2_].

d Effective ionic radii.

**Scheme 3:**
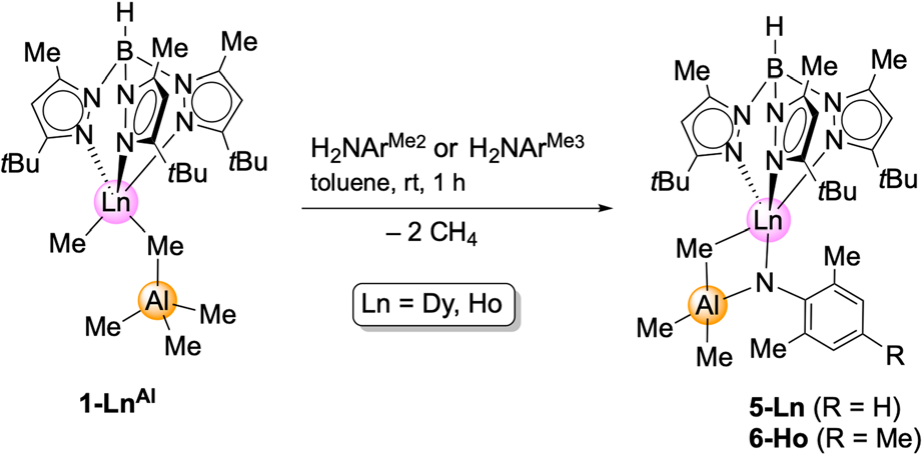
Synthesis of Lewis acid stabilized, bimetallic imides Tp^*t*Bu,Me^Ln(NAr^Me2^)(μ_2_-MeAlMe_3_) (5-Ln; Ln = Dy, Ho) and Tp^*t*Bu,Me^Ho(NAr^Me3^)(μ_2_-MeAlMe_3_).

In contrast, and as pointed out previously, the reaction of the primary aniline H_2_NAr^Me_2_^ (Ar^Me_2_^ = C_6_H_3_Me_2_-2,6) with the aluminium congeners Tp^*t*Bu,Me^LnMe(AlMe_4_) (1-Ln^Al^) implies the formation of trimethylaluminium-stabilized imide complexes.^12^ These complexes cannot be converted into unsupported terminal rare-earth-metal imide complexes by applying Lewis bases such as 1,4-dioxane, pyridine, DMAP, or TMEDA (*N*,*N*,*N*′,*N*′-tetramethylethylenediamine). Complex 1-Dy^Al^ is accessible from Dy(AlMe_4_)_3_ (ref. 24) and H[Tp^*t*Bu,Me^],^22^ in analogy to the yttrium and holmium complexes reported previously.^20^ In order to probe the effect of the substituents on the aniline, compounds 1-Ln^Al^ (Ln = Dy, Ho) were reacted with H_2_NAr^iPr^, H_2_NAr^Me2^, and H_2_NAr^Me3^ (Ar^Me3^ = C_6_H_2_Me_3_-2,4,6) (Scheme 3).

**Fig. 3:**
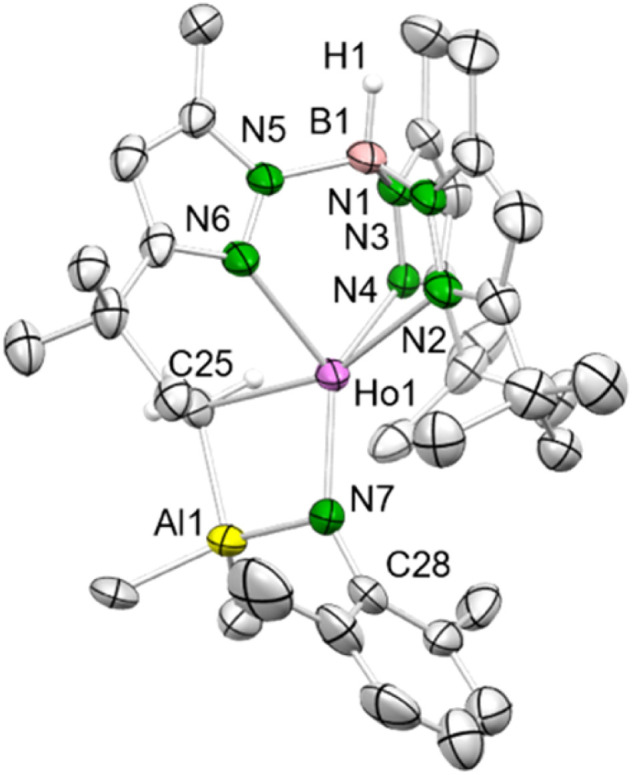
Crystal structure of 5-Ho. All atoms are represented by atomic displacement ellipsoids set at 50% probability. Solvent molecules, and hydrogen atoms except for that of B–H are omitted for clarity. Selected interatomic distances (Å) and angles (°): Ho1–N7 2.116(4), Ho1–C25 2.549(5), Al1–C25 2.105(6); Ho1–N7–C28 146.7(4) (for isostructural 5-Dy and 6-Ho and further metrics, see ESI†).

Contrary to the reaction with the weaker coordinated trimethylgallium in complexes 1-Ln^Ga^, the 1-Ln^Al^/H_2_NAr^iPr^ reaction was inconclusive. Like in the case of yttrium,^12^ the sterically less demanding anilines gave the trimethylaluminium-stabilized imides Tp^*t*Bu,Me^Ln(μ_2_-NAr^Me2^)AlMe_3_ (5-Ln: Ln = Dy, Ho) and Tp^*t*Bu,Me^Ho(μ_2_-NAr^Me3^)AlMe_3_ (6-Ln). The coordinated trimethylaluminium is not removable, neither under vacuum, nor with Lewis bases (*e.g.*, DMAP, THF). Presumably, after the first methane elimination and the coordination of the primary amido functionality to the metal centre, one of the methyl groups at the [μ_2_-MeAlMe_3_] unit abstracts the second amido proton *via* release of another molecule of methane, resulting in 5-Ln. Compounds 5-Ln are insoluble in aliphatic solvents, but dissolve in aromatic and polar solvents. The isostructural complexes crystallize in different space groups (5-Dy: monoclinic, *P*2_1_/*n*; 5-Ho: triclinic, *P*1̄; 6-Ho: monoclinic, *Cc*; Fig. 3, S10 and S11†).

The bulky Tp^*t*Bu,Me^ ligand coordinates again in the κ^3^ fashion (*N*, *N*′, *N*′′) with interatomic Ln–N distances in the range of 2.423(2)–2.492(2) Å (5-Dy), 2.407(4)–2.437(4) Å (5-Ho) and 2.394(3)–2.454(3) Å (6-Ho). The central metal ion is pentacoordinate and the Ln–N_imido_–C_ipso_ angle is strongly bent (5-Dy: 146.68(17)°; 5-Ho: 146.7(4)°; 6-Ho: 151.0(2)°), which is due to the interaction with the Lewis acid trimethylaluminium. Hence, the electronic situation differs considerably compared to the terminal rare-earth-metal imide complexes 4-Ln (Ln = Dy, Ho) as electron density of the imido nitrogen is shifted to the empty p orbitals of the aluminium ion. This is also reflected in the Ln–N_imido_ bonds of 5-Ln and 6-Ho which are elongated by *ca.* 0.1 Å (5-Dy: 2.129(2) Å; 5-Ho: 2.116(4) Å; 6-Ho: 2.116(4) Å), matching those in Tp^*t*Bu,Me^Y(μ_2_-NAr^Me^)AlHMe_2_ (Y–N_imido_: 2.133(2) Å; Y–N_imido_–C_ipso_: 145.1(2)°).^25^ Further structural comparison with the similar holmium imides Tp^*t*Bu,Me^Ho(μ_2_-NR)AlMe_3_ (R = *t*Bu, adamantyl) clearly indicate a more pronounced Ln–N_imido_ interaction for the latter (Ho–N_imido_: 2.083(2), 2.087(2) Å; Ho–N_imido_–C_ipso_: 140.4(2), 140.2(1)°).^20*a*^ Other Lewis acid supported monomeric rare-earth-metal imide complexes include Mindiola's (PNP)Sc(μ_2_-NAr^iPr^)(μ_2_-Me)AlMe_2_ [PNP=N(2-P-(CHMe_2_)_2_-4-methylphenyl)_2_] (ref. 26) or Tp^*t*Bu,Me^Ln(NAr^iPr^)(MMe_3_) (Ln = Ce, Nd, Sm; M = Al, Ga) from our group.^6*c*^

### Probing donors other than DMAP

**Scheme 4:**
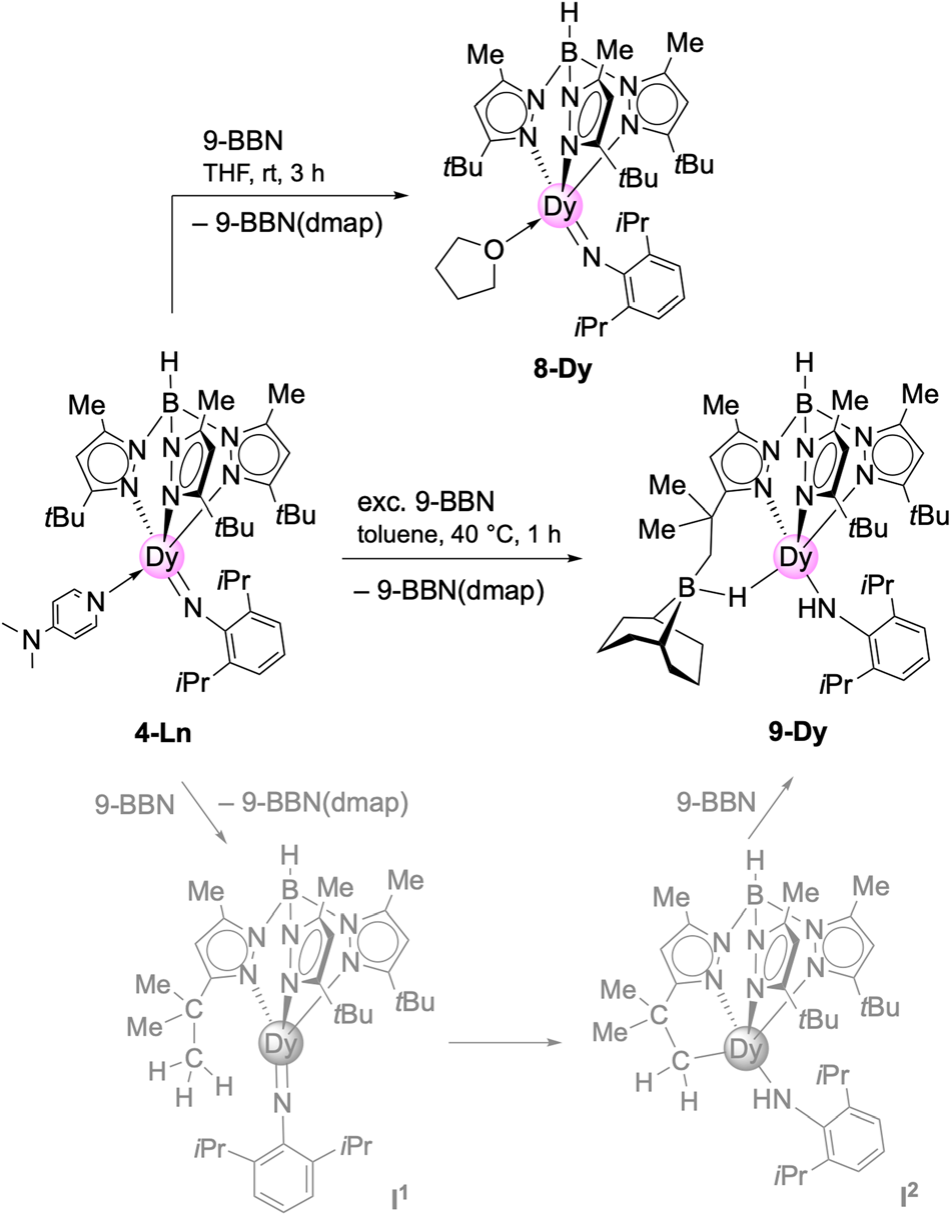
Reactivity of Tp^*t*Bu,Me^Dy(NAr^iPr^)(dmap) (4-Dy) (Ln = Y, Dy) towards Lewis acid 9-BBN in THF and toluene.

*N*,*N*-Dimethyl-4-aminopyridine (DMAP) emerged as a most valuable donor for forcing the elimination of methane *via* inner-sphere deprotonation of the primary amido species in 2-Ln-type complexes. In order to assess the impact of the donor molecule on the Ln–N_imido_ bonding, the implementation of other donor ligands was examined. In general, donor ligands might be introduced according to Scheme 1 promoting methane elimination (route A) or *via* post-imide-synthesis exchange (Scheme 4). Since imido ligand formation according to route A could not be achieved with ethereal donors such as OEt_2_ or THF, complex 2-Dy was treated with N-donors TMEDA (tetramethylethylenediamine), TMPDA (tetramethylpropane-1,3-diamine) and acetonitrile. While the potentially bidentate diamines did not undergo any reaction yielding only in the isolation of the starting compounds, acetonitrile formed the insertion complex Tp^*t*Bu,Me^Dy[NC(Me)_2_](HNAr^iPr^) (7-Dy, Scheme 2). It was previously shown that acetonitrile does react with rare-earth-metal alkyls either *via* C–H-bond activation/deprotonation or insertion.^27–29^ While the product of the deprotonation reaction (C_5_Me_5_)_2_La[CH(SiMe_3_)_2_]/CH_3_CN was structurally characterized as [(C_5_Me_5_)_2_La(μ-CH_2_CN)]_2_,^27^ the insertion product of the reaction (C_5_Me_5_)_2_ScCH_3_/CH_3_CN was only spectroscopically analyzed as (C_5_Me_5_)_2_Sc[NC(Me)_2_].^28^ The SCXRD study of five-coordinate 7-Dy features distinct bonding behaviour of the primary amido and the dimethyliminato ligand (Dy–N: 2.219(3) *versus* 2.148(3) Å; Dy–N–C: 156.3(3) *versus* 165.3(3)°) (Fig. 2). Thus, the dimethyliminato coordination compares to that of imidazolin-2-iminato complexes like LY(CH_2_SiMe_3_)_2_(thf)_2_ (L = 1,3-bis(2,6-diisopropylphenyl)imidazoline-2-iminato; Y–N, 2.1255(13) Å, Y–N–C, 176.85(12)° or LYCl_2_(thf)_3_ Y–N, 2.1278(18) Å, Y–N–C, 174.35(16)°) supposedly featuring very short Ln–N_iminato_ bonds.^30,31^

The post-synthesis exchange approach (route 2, Scheme 4) was probed with 4-Dy and the strong Lewis acid 9-borabicyclo[3.3.1]nonane (9-BBN) in THF.^11^ Accordingly, the equimolar reaction led to the displacement of DMAP and coordination of one THF molecule to the dysprosium centre in Tp^*t*Bu,Me^Dy(NAr^iPr^)(thf) (8-Dy, Scheme 4). Like the dmap adduct, 8-Dy is soluble in toluene and THF, but insoluble in aliphatic solvents. It crystallized in the orthorhombic space group *Pna*2_1_ and shows the same κ^3^ coordination of the Tp*^t^*^Bu,Me^ ligand as complex 4-Dy before (Dy–N_pz_: 2.465(4)–2.510(4)/2.450(3)–2.539(4) Å) (Fig. 2). As the coordination number did not change, the angle of the imido functionality stayed nearly the same (Dy–N_imido_–C_ipso_: 165.2(3)/166.9(3)°) as detected for 4-Dy. However, the Dy–N_imido_ distance of 2.004(4)/2.008(3) Å) in 8-Dy appears to be slightly shorter compared to 4-Dy (Table 1) which can be attributed to the weaker donor properties of the thf ligand.

The 9-BBN-promoted donor exchange was previously introduced by Chen, revealing that prior activation of L^1^Sc(NAr^iPr^)(dmap) (L^1^ = [Ar^iPr^NC(Me)CHC(Me)N(CH_2_)_2_NMe_2_]) with 9-BBN led to abstraction of the donor molecule DMAP.^11^ The emerging donor-free imide intermediate [L^1^Sc(NAr^iPr^)] could be trapped with THF to afford L^1^Sc(NAr^iPr^)(thf) featuring also a shorter Sc–N_imido_ bond than the DMAP adduct (Table 1: 1.852(4) *versus* 1.881(8) Å). It was also mentioned that the direct synthesis of the THF adduct L^1^Sc(NAr^iPr^)(thf) is not possible by thermolysis of the mixed methyl/amido scandium complex in THF, which is the same case for 8-Dy as well.^2^ In contrast, terminal imides Tp^*t*Bu,Me^Ln(NAr^iPr^)(thf)_2_ of the larger rare-earth metals can be obtained directly according to the donor(THF)-assisted methylidene → imido transformation (Scheme 1 and Table 1).^6*c*^

**Fig. 4:**
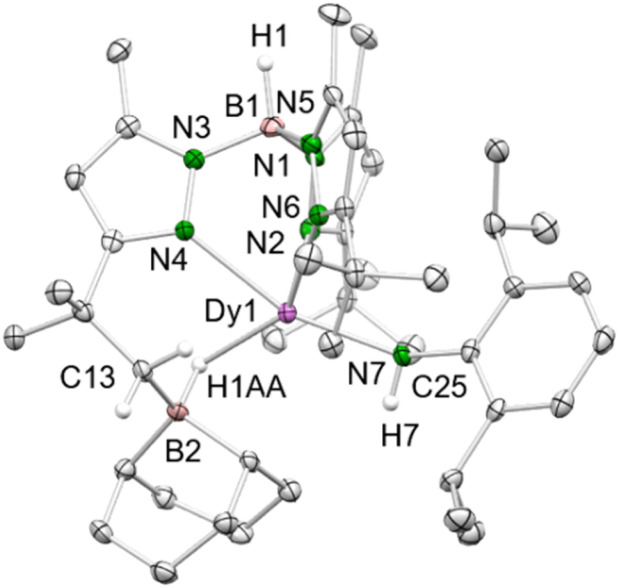
Crystal structure of 9-Dy. All atoms are represented by atomic displacement ellipsoids set at 50% probability. Solvent molecules and hydrogen atoms except for those of B–H and N–H are omitted for clarity. Selected bond distances (Å) and angels (°) for 9-Dy: Dy1–N7 2.237(3), Dy 1–N2 2.452(3), Dy 1–N4 2.405(3), Dy 1–N6 2.461(3), Dy 1–B2 2.703(4); Dy1–H1AA 2.17(3), B2–H1AA 1.23(3); Dy1–N7–C35 143.4(2), N7–Dy1–B2 117.06(11), N7–Dy1–N4 161.07(10), N7–Dy1–N2 93.18(10), N7–Dy1–N6 92.04(10).

Treatment of 4-Dy with excess of 9-BBN in toluene gave the mixed primary amido/hydroborato complex 9-Dy (Scheme 4 and Fig. 4). Again, this is in line with the observation made by Chen with the system L^2^Sc(NAr^iPr^)/9-BBN (L^2^ = [Ar^iPr^NC(Me)CHC(Me)N(CH_2_)_2_N(CH_2_)_2_NMe_2_]).^32^ Correspondingly, it can be hypothesized that initially the strong Lewis acid 9-BBN displaces all of the coordinated DMAP, rendering a highly reactive donor-free terminal imide [Tp^*t*Bu,Me^Dy(NAr^iPr^)] (Scheme 4, intermediate I^1^, lower trace). Subsequent 1,2-addition of a *t*Bu methyl group (C–H-bond activation) across the highly reactive Dy–N_imido_ bond of transient species I^1^ reforms the primary amido ligand along with a 5-membered metallacycle in I^2^. Then, the highly nucleophilic alkyl attached to the dysprosium attacks a second molecule of 9-BBN to afford the alkylhydroborato moiety. The resulting Dy–N_amido_–C_ipso_ angle (143.4(2)°) and the Dy–N_amido_ distance (2.237(3) Å) of 9-Dy are comparable to the dysprosium amide complexes discussed beforehand. The Dy–B distance of 2.703(4) Å is in the range of the Y–B distances in [(Me_3_Si)_2_NC(NiPr)_2_]Y[μ-H(μ-Et)_2_BEt]_2_(thf)_2_ (2.658(4) and 2.671(4) Å)^33^ and (C_5_Me_5_)_2_YH(9-BBN) (2.767(6) Å),^34^ but longer than those observed in (*t*Bu_4_Carb)Dy(BH_4_)_2_(thf) (2.473(2) and 2.487(2) Å).^35^

In Chen's system [L^2^Sc(NAr^iPr^)]/9-BBN the respective C(sp^3^)–H bond borylation took place at the [–CH_2_NMe_2_] side arm of the ancillary nacnac ligand (Sc–H_hydrido_, 2.01(3) Å).^32^ Chen also reported on the reaction of L^1^Sc(NAr^iPr^)(dmap) with three equivalents of 9-BBN which led to mixed boroamido/hydroborato complex L^1^Sc[NAr^iPr^(9-BBN–H)](9-BBN + H) (Sc–H_hydrido_, 1.89 Å and 1.20 Å).^11^

**Fig. 5:**
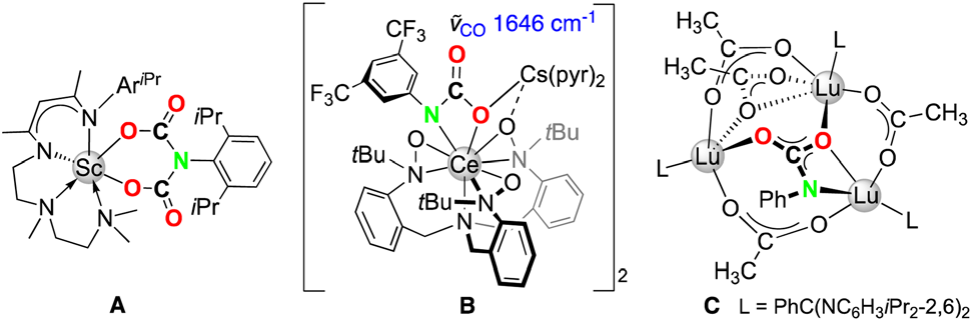
Structurally authenticated CO_2_-insertion products A,^17*a*^B,^38^ and C (ref. 17*d*) emerged from rare-earth-metal imides.

Preliminary studies on the reactivity of terminal imide complex 4-Dy towards carbon dioxide (1 bar) in toluene at ambient temperature clearly indicate CO_2_ insertion into the Dy–N_imido_ bond and, hence, carbamate formation in product 10-Dy. The DRIFT spectrum of 10-Dy revealed typical carbonyl vibrations at 1701 cm^−1^ and 1641 cm^−1^ assigned to asymmetric and symmetric C=O stretching vibrations (Fig. S17†). Moreover, the B–H vibration of 4-Dy at 2557 cm^−1^, typical for the terminal B–H stretch of the Tp^*t*Bu,Me^ ligand when coordinated in a tridentate fashion,^36^ changed to 2431 cm^−1^ in 10-Dy. Such a dramatic change of the position of the B–H band can be ascribed to a κ^3^ → κ^2^ coordination switch of the Tp^*t*Bu,Me^ ligand, likely involving a Dy⋯H–B interaction.^37^ Unfortunately, crystals of 10-Dy suitable for SCXRD analysis could not be obtained, but 10-Dy can be tentatively assigned as the dicarboxylate species [Tp^*t*Bu,Me^Dy{(O_2_C)_2_NAr^iPr^}] on the basis of the double CO_2_-insertion chemistry of the terminal scandium imides L^2^Sc(NAr^iPr^) (A, Fig. 5)^17*a*^ and (BPz_2_Py_3_)Sc(NAr^iPr^) (*ν̃* = 1685 and 1632 cm^−1^).^13^ For further comparison, mono insertion of carbon dioxide was observed for the Lu_3_(μ_3_-NPh) moiety of the trinuclear cluster [L^3^Lu_3_(μ_2_-Me)_3_(μ_3_-Me)(μ_3_-NPh)] (L^3^ = PhC(NC_6_H_3_iPr_2_-2,6; C, Fig. 5)^17*d*^ and the alkali-metal stabilized Ce(iv)–N_imido_ bond of [(TriNOx)Ce(NAr^CF_3_^)][Cs(2.2.2-cryptand)] (B, Fig. 5).^38^

## Conclusion

The successful application of the donor-assisted intramolecular primary amido deprotonation protocol towards terminal imides of the rare-earth metals yttrium, dysprosium, and holmium depends on two crucial factors: first, the kinetic stabilization of the targeted imide *via* appropriate steric shielding of the ancillary ligand, herein supplied by the bulky Tp^*t*BuMe^ scorpionate ligand; second, sufficient Brønsted acidity and steric demand of the employed primary amine, herein provided by H_2_NAr^iPr^ (Ar^iPr^ = C_6_H_3_iPr_2_-2,6). With these key factors in mind, it was possible to generate a series of terminal rare-earth-metal imides Tp^*t*Bu,Me^Ln(NAr^iPr^)(dmap) for mid-sized to small rare-earth metals. Their reactivity parallels that of terminal scandium imides, as revealed by treatment with the strong Lewis acid 9-borabicyclo[3.3.1]nonane (9-BBN) and the heteroallene CO_2_. These reactions revealed effective DMAP/THF donor exchange and CO_2_ insertion into the Ln–N_imido_ bond (carbamate formation). The reactivity studies also include the isolation and structural characterization of the bis(amido) species Tp^*t*Bu,Me^Ho(NAd)_2_ (Ad = adamantyl) and dimethyliminate complex Tp^*t*Bu,Me^Dy[NC(Me)_2_](HNAr^iPr^).

## Data availability

Experimental, spectroscopic and structural data supporting this article have been uploaded as part of the ESI. Crystallographic data for all compounds have been deposited at the CCDC under 2312212–2312226, and can be obtained from https://www.ccdc.cam.ac.uk/structures/.

## Author contributions

TR, synthesis and characterization of compounds, writing original draft; DS, synthesis and characterization of compound 1-Ho^Ga^, editing original draft; CM-M, crystallography, editing original draft; RA, conceptualization, supervision, writing and project administration, funding acquisition.

## Conflicts of interest

There are no conflicts to declare.

## Supplementary Material

SC-015-D3SC06584G-s001

SC-015-D3SC06584G-s002
