# Supplementary material for: Terminal dysprosium and holmium organoimides
Source: Chem Sci. 2024 Jan 22;15(10):3562–70. doi: 10.1039/d3sc06584g (PMC10915843; doi:10.1039/d3sc06584g)
Supplement: SC-015-D3SC06584G-s001 [file SC-015-D3SC06584G-s001.pdf]

Electronic Supplementary Material (ESI) for Chemical Science.
This journal is © The Royal Society of Chemistry 2024

Supporting Information

Terminal Dysprosium and Holmium Organoimides

Theresa E. Rieser, Dorothea Schädle, Cäcilia Maichle-Mössmer, and Reiner Anwander*

Institut für Anorganische Chemie, Eberhard Karls Universität Tübingen, Auf der Morgenstelle 18, 72076 Tübingen, Germany

*E-mail for R.A.: reiner.anwander@uni-tuebingen.de.

Table of Contents

Experimental Section..........S3

General Considerations..........S3

General procedure of **1-Ln**..........S3

Synthesis procedures of **1-Ln-10-Ln**..........S3

Figure S1. Crystal structure of $Ho(Ga(CH_3)_4)_3$..........S8

Figure S2. Crystal structure of $Dy(Ga(CH_3)_4)_3$..........S8

Figure S3. Crystal structure of **1-Ho^{Ga}**..........S9

Figure S4. Crystal structure of **2-Dy**..........S10

Figure S5. Crystal structure of **2-Ho**..........S11

Figure S6. Crystal structure of **3-Ho**..........S12

Figure S7. Crystal structure of **4-Y**..........S13

Figure S8. Crystal structure of **4-Dy**..........S14

Figure S9. Crystal structure of **4-Ho**..........S15

Figure S10. Crystal structure of **5-Dy**..........S16

Figure S11. Crystal structure of **5-Ho**..........S17

Figure S12. Crystal structure of **6-Ho**..........S18

Figure S13. Crystal structure of **7-Dy**..........S19

Figure S14. Crystal structure of **8-Dy**..........S20

Figure S15. Crystal structure of **9-Dy**..........S21

Tables S1. Crystallographic data for **1-Dy**, **2-Dy** and **2-Ho**..........S22

Tables S2. Crystallographic data for **4-Y**, **4-Dy**, and **4-Ho**..........S23

Tables S3. Crystallographic data for **5-Dy**, **5-Ho**, and **6-Ho**..........S24

Tables S4. Crystallographic data for **7-Dy**, **9-Dy**, and **3-Ho**..........S25

Tables S5. Crystallographic data for **8-Ho**, $Ho[Ga(CH_3)_4]_3$ and $Dy[Ga(CH_3)_4]_3$..........S26

Crystallography and Crystal Structure Determinations..........S27

Figure S16. DRIFT spectrum of **4-Dy**..........S28

Figure S17. DRIFT spectrum of **10-Dy**..........S28

Figure S18. DRIFT spectrum of HTp^{tBu,Me} S28

NMR Spectroscopy S29

Figure S19. ^{1}H NMR spectrum of **1-Y^{Ga}** S29

Figure S20. ^{1}H NMR spectrum of **2-Y** S29

Figure S21. ^{1}H NMR spectrum of **4-Y** S30

References S31

Experimental Section

General Considerations. All manipulations were performed under rigorous exclusion of air and moisture, using standard Schlenk, high-vacuum, and glovebox techniques (MB Braun MB200B; < 0.1 ppm O_2, < 0.1 ppm H_2O, argon atmosphere). The solvents *n*-hexane, *n*-pentane, diethyl ether, toluene and tetrahydrofuran (THF) were purified using Grubbs columns (MBraun SPS, solvent purification system). Tetrahydrofuran was stored over freshly activated molecular sieves (3 Å). 2,6-Di*iso*propylaniline (H_2NAr^{iPr}, 97%, Sigma-Aldrich), 2,6-dimethylaniline (H_2NAr^{Me}, 99%, Sigma-Aldrich), and 2,4,6-trimethylaniline (H_2NAr^{Mes}, 98%, Sigma-Aldrich) were dried over CaH_2 and purified by distillation. 1-Adamantylamine (H_2NAd, 97%, Sigma-Aldrich) was used without further purification. All solvents and reagents were stored inside a glovebox. C_6D_6 and toluene-d_8 were obtained from *Aldrich* or *eurisotop*, degassed, dried over Na for 24 h and filtered. $HTp^{tBu,Me}$ and $KTp^{tBu,Me}$ ($Tp^{tBu,Me}$ = hydrotris(3-tert-butyl-5-methylpyrazol)borate) were synthesized by a modification of the published procedure of $HTp^{tBu,Ph}$.[1] $Ln(AlMe_4)_3$ and $Ln(GaMe_4)_3$ (Ln = Y, Dy, Ho) were synthesized according to literature procedures.[2,3] The NMR spectra of air and moisture sensitive compounds were recorded by using *J. Young*-valved NMR tubes on a *Bruker AVII+400* spectrometer (^{1}H: 400.13 MHz) and AV500 or *Bruker* AVII+500 (5 mm BBO, ^{1}H: 500.13 Hz; ^{13}C: 125.77 MHz). ^{1}H NMR resonances are referenced to solvent residual resonances and reported in *parts per million*, relative to tetramethylsilane (TMS). Coupling constants are given in Hertz. IR spectra were recorded on a *NICOLET 6700 FTIR* spectrometer with a DRIFT cell (KBr window), and the samples were prepared in a glovebox and mixed with KBr powder. Elemental analyses were performed on an *Elementar Vario Micro Cube*.

General procedure for the synthesis of $Tp^{tBu,Me}LnMe(GaMe_4)$ (1-Ln^{Ga}). To a solution of one equivalent $Ln(GaMe_4)_3$ in *n*-pentane (3 mL) a solution of one equivalent $HTp^{tBu.Me}$ in *n*-pentane (3 mL) was slowly added. The mixture was stirred for 15 minutes. After formation of a precipitate, the solution was decanted, and the remaining powder was washed with *n*-pentane (3x 5 mL).

$Tp^{tBu,Me}YMe(GaMe_4)$ (1-Y^{Ga}). Following the procedure described above, $Y(GaMe_4)_3$ (100 mg, 0.210 mmol) and $HTp^{tBu.Me}$ (89.3 mg, 0.210 mmol) yielded **1-Y^{Ga}** as a white precipitate (42 mg, 0.0639 mmol, 30%). ^{1}H NMR (400 MHz, C_6D_6, 26° C): δ 5.60 (s, 3H, pz-*H*), 4.57 (br, 1H, B*H*), 2.01 (s, 9H, pz-C*H*$_3$), 1.42 (s, 27H, pz-C(C*H*$_3$)$_3$) – 0.09 (s, 15H, Y/Ga(C*H*$_3$)) ppm. Elemental analysis (%) calculated for $BN_6C_{29}H_{55}GaY$ (657.24 g/mol): C 53.00, H 8.44, N 12.79; found: C 53.15, H 8.23, N 12.65.

Tp^{tBu,Me}DyMe(GaMe$_4$) (1-Dy^{Ga}). Following the procedure described above, Dy(GaMe$_4$)$_3$ (198 mg, 0.359 mmol) and HTp$^{tBu.Me}$ (152 mg, 0.359 mmol) yielded **1-Dy^{Ga}** as a yellow precipitate (221 mg, 0.302 mmol, 84%). Single crystals suitable for XRD analysis formed from a toluene solution. Elemental analysis (%) calculated for $BN_6C_{29}H_{55}GaDy$ (730.83 g/mol): C 47.66, H 7.59, N 11.50; found: C 47.87, H 7.53, N 11.34.

Tp^{tBu,Me}HoMe(GaMe$_4$) (1-Ho^{Ga}). Following the procedure described above, Ho(GaMe$_4$)$_3$ (111 mg, 0.20 mmol) and HTp$^{tBu.Me}$ (85 mg, 0.20 mmol) yielded **1-Ho^{Ga}** as a pink precipitate (79 mg, 0.108 mmol, 39%). ^{1}H NMR (500 MHz, toluene-*d*$_8$, 26 °C): δ 101.83, 50.92, –8.78, –15.58, –152.38, –177.58 ppm. DRIFT (KBr): 2964 s, 2906 s, 2877 m, 2806 ww, 2564 w (B-H), 1539 vs, 1489 w, 1473 m, 1464 m, 1431 vs, 1382 w, 1365 m, 1351 s, 1326 m, 1240 w, 1202 m, 1191 m, 1165 vs, 1141 m, 1065 s, 1027 m, 799 s, 763 s, 731 w, 682 s, 643 m, 584 w, 524 m, 471 w, 411 m cm^{-1}. Elemental analysis calcd (%) for $C_{29}H_{55}BGaHoN_6$: C 47.50, H 7.56, N 11.46; found: C 47.32, H 7.92, N 11.21.

General procedure for the synthesis of Tp^{tBu,Me}LnMe(HNC$_6$H$_3$*i*Pr$_2$-2,6) (2-Ln). To a solution of one equivalent **1-Ln** in toluene (3 mL) a solution of one equivalent H$_2$NC$_6$H$_3$*i*Pr$_2$-2,6 in toluene (3 mL) was slowly added. The mixture was stirred for one hour at ambient temperature. After the solvent was removed under vacuum, the powder was washed with *n*-hexane (3x 5 mL).

Tp^{tBu,Me}YMe(HNC$_6$H$_3$*i*Pr$_2$-2,6) (2-Y). Following the procedure described above, **1-Y** (563 mg, 0.857 mmol) and H$_2$NC$_6$H$_3$*i*Pr$_2$-2,6 (152 mg, 0.857 mmol) yielded **2-Y** as a white precipitate (467 mg, 0.664 mmol, 77%). ^{1}H NMR (400 MHz, C_6D_6, 26 °C): δ 7.08 (d, 2H, $^3J_{HH}$ 7.52 Hz, *m*-Ar*H*), 6.77 (t, 1H, $^3J_{HH}$ 7.36 Hz, *p*-Ar*H*), 5.56 (s, 3H, pz-*H*), 4.87 (s, 1H, N*H*), 2.87 (br, 2H, *i*Pr-*H*), 2.13 (s. 9H, pz-C*H*$_3$), 1.39 (s, 27H, pz-C(C*H*$_3$)$_3$), 1.13 (d, 12H, $^3J_{HH}$ 6.43 Hz, *i*Pr-C*H*$_3$), 0.46 (d, 3H, $^2J_{YH}$ 1.74 Hz, Y-C*H*$_3$) ppm. Elemental analysis (%) calculated for $BN_7C_{37}H_{61}Y$ (703.66 g/mol): C 63.16, H 8.74, N 13.93; found: C 63.35, H 8.58, N 13.72.

Tp^{tBu,Me}DyMe(HNC$_6$H$_3$*i*Pr$_2$-2,6) (2-Dy). Following the procedure described above, **1-Dy** (70 mg, 0.0958 mmol) and H$_2$NC$_6$H$_3$*i*Pr$_2$-2,6 (15.3 mg, 0.0862 mmol) yielded **2-Dy** as a yellow precipitate (63 mg, 0.0811 mmol, 85%). Single crystals suitable for XRD analysis formed from a toluene solution. Elemental analysis (%) calculated for $BN_7C_{37}H_{61}Dy$ (777.25 g/mol): C 57.18, H 7.91, N 12.61; found: C 57.30, 7.77, N 12.40.

Tp^{tBu,Me}HoMe(HNC$_6$H$_3$*i*Pr$_2$-2,6) (2-Ho). Following the procedure described above, **1-Ho** (100 mg, 0.136 mmol) and H$_2$NC$_6$H$_3$*i*Pr$_2$-2,6 (21.8 mg, 0.123 mmol) yielded **2-Ho** as a yellow precipitate (87 mg, 0.112 mmol, 82%). Single crystals suitable for XRD analysis formed from a toluene solution. Elemental analysis (%) calculated for BN$_7$C$_{37}$H$_{61}$Ho (779.68 g/mol): C 57.00, H 7.89, N 12.58; found: C 57.28, H 6.87, N 12.44.

Tp^{tBu,Me}Ho(HNC$_{10}$H$_{15}$)$_2$ (3-Ho). To a solution of **1-Ho** (30.0 mg, 0.0409 mmol) in toluene (2 mL) a solution of H$_2$NC$_{10}$H$_{15}$ (5.57 mg, 0.0368 mmol) in toluene (3 mL) was added and stirred for 15 minutes at – 35 °C. After the solvent was removed under vacuum and the powder was washed with *n*-hexane (3x 5 mL), single crystals suitable for XRD analysis formed from a toluene solution. Elemental analysis (%) calculated for BN$_8$C$_{44}$H$_{72}$Ho (888.86 g/mol): C 59.46, H 8.17, N 12.61; found: C 59.28 H 8.09, N 12.54.

General procedure for the synthesis of Tp^{tBu,Me}Ln(NC$_6$H$_3$*i*Pr$_2$-2,6)(dmap) (4-Ln). To a solution of one equivalent **2-Ln** in toluene (3 mL) a solution of one equivalent DMAP in toluene (3 mL) was slowly added. The mixture was stirred for one hour at ambient temperature. After the solvent was removed under vacuum, the powder was washed with *n*-hexane (3x 5 mL).

Tp^{tBu,Me}Y(NC$_6$H$_3$*i*Pr$_2$-2,6)(dmap) (4-Y). Following the procedure described above, **2-Y** (330 mg, 0.469 mmol) and DMAP (57.3 mg, 0.469 mmol) yielded **4-Y** as a beige precipitate (279 mg, 0.345 mmol, 74%). Single crystals suitable for XRD analysis formed from a toluene solution. ^{1}H NMR (400 MHz, C$_6$D$_6$, 26 °C): δ 8.47 (d, 1H, $^3J_{HH}$ 6.30 Hz, DMAP, H_{ortho}), 8.01 (s br, 1H, DMAP, H_{ortho}), 7.04 (d, 2H, $^3J_{HH}$ 7.42 Hz, *m*-Ar*H*), 6.71 (t, 1H, $^3J_{HH}$ 7.27 Hz, *p*-Ar*H*), 6.35 (dd, 1H, ^{3}J$_{HH}$ 6.28 Hz, ^{4}J$_{HH}$ 2.71 Hz, DMAP, H_{meta}), 5.72 (s, 3H, pz-*H*), 5.53 (s br, 1 H, DMAP, H_{meta}), 2.79 (br, 2H, *i*Pr-*H*), 2.51 (s, 6H, N(C*H*$_3$)$_2$) 2.24 (s. 9H, pz-C*H*$_3$), 1.39 (s, 27H, pz-C(C*H*$_3$)$_3$), 0.99 (d, 12H, $^3J_{HH}$ 6.93 Hz, *i*Pr-C*H*$_3$) ppm. Elemental analysis (%) calculated for BN$_9$C$_{43}$H$_{67}$Y (809.79 g/mol): C 63.78, H 8.34, N 15.57; found: C 64.35, H 8.95, N 14.58.

Tp^{tBu,Me}Dy(NC$_6$H$_3$*i*Pr$_2$-2,6)(dmap) (4-Dy). Following the procedure described above, **2-Dy** (321 mg, 0.413 mmol) and DMAP (50.5 mg, 0.413 mmol) yielded **4-Dy** as a yellow precipitate (109 mg, 0.123 mmol, 30%). Single crystals suitable for XRD analysis formed from a toluene solution. $\tilde{\nu}_{max}$ = 3056 vw, 3023 vw, 2960 vs, 2930 s, 2862 m, 2557 (B-H), 1612 vs, 1582 vs, 1542 vs, 1489 w, 1463 m, 1427 vs, 1399 s, 1351 s, 1329 vs, 1267 s, 1230 m, 1199 m, 1179 s, 1138 w, 1065 s, 1006 s, 990 m, 906 m, 848 w, 802 m, 790 m, 770 m, 739 m, 683 w, 645 m, 541 vw, 515 vw, 441 vw, 415 vw cm^{-1}. Elemental analysis (%) calculated for BN$_9$C$_{43}$H$_{67}$Dy (883.38 g/mol): C 58.47, H 7.65, N 14.27; found: C 60.04, 7.54, N 13.49.

Tp^{tBu,Me}Ho(NC$_6$H$_3$*i*Pr$_2$-2,6)(dmap) (4-Ho). Following the procedure described above, **2-Ho** (177 mg, 0.227 mmol) and DMAP (27.7 mg, 0.227 mmol) yielded **4-Ho** as an orange precipitate (78.3 mg, 0.0884 mmol, 39%). Single crystals suitable for XRD analysis formed from a toluene solution. Elemental analysis (%) calculated for $BN_9C_{43}H_{67}Ho$ (885.81 g/mol): C 58.31, H 7.62, N 14.23; found: C 58.04, H 7.61, N 13.79.

General procedure for the synthesis of Tp^{tBu,Me}Ln(NC$_6$H$_3$*i*Pr$_2$-2,6)(AlMe$_3$) (5-Ln). To a solution of Tp^{tBu,Me}DyMe(AlMe$_4$) (**1-Ln^{Al}**) in toluene (3 mL) a solution of an equimolar amount of $H_2NC_6H_3Me_2$-2,6 in toluene (3 mL) was slowly added. The mixture was stirred for one hour at ambient temperature. After the solvent was removed under vacuum, the powder was washed with *n*-hexane (3x 5 mL).

Tp^{tBu,Me}Dy(NC$_6$H$_3$Me$_2$-2,6)(AlMe$_3$) (5-Dy). Following the procedure described above, Tp^{tBu,Me}DyMe(AlMe$_4$) (**1-Dy^{Al}**, 80.3 mg, 0.0964 mmol) and $H_2NC_6H_3Me_2$-2,6 (11.7 mg, 0.0964 mmol) yielded **5-Dy** as a yellow precipitate (27.8 mg, 0.0358 mmol, 37%). Single crystals suitable for XRD analysis formed from a toluene solution.

Tp^{tBu,Me}Ho(NC$_6$H$_3$Me$_2$-2,6)(AlMe$_3$) (5-Ho). Following the procedure described above, **1-Ho^{Al}** (121 mg, 0.155 mmol) and $H_2NC_6H_3Me_2$-2,6 (18.8 mg, 0.155 mmol) yielded **5-Ho** as a yellow precipitate (37.5 mg, 0.0481 mmol, 31%). Single crystals suitable for XRD analysis formed from a toluene solution. Elemental analysis (%) calculated for $BN_7C_{35}H_{58}HoAl$ (779.62 g/mol): C 53.92, H 7.50, N 12.58; found: C 54.04, H 7.60, N 12.04.

Tp^{tBu,Me}Ho(NC$_6$H$_2$Me$_3$-2,4,6)(AlMe$_3$) (6-Ho). To a solution of Tp^{tBu,Me}HoMe(AlMe$_4$) (**1-Ho^{Al}**) (100 mg, 0.136 mmol) in toluene (4 mL) a solution of $H_2NC_6H_3Me_3$-2,4,6 (16.5 mg, 0.122 mmol) in toluene (3 mL) was added and stirred for 1 h at ambient temperature. After the solvent was removed under vacuum and the powder was washed with *n*-hexane (3x 5 mL), single crystals suitable for XRD analysis formed from a toluene solution.

Tp^{tBu,Me}Dy(HNC$_6$H$_3$*i*Pr$_2$-2,6)(NC(CH$_3$)$_2$) (7-Dy). To a solution of **2-Dy** (200 mg, 0.257 mmol) in toluene (6 mL) acetonitrile (21 mg, 0.512 mmol) was added. The solution was stirred at ambient temperature overnight. After the solvent was removed under vacuum and the yellow powder was washed with *n*-hexane (3x 5 mL), single crystals suitable for XRD analysis formed from a toluene solution.

Tp^{tBu,Me}Dy(NC$_6$H$_3$*i*Pr$_2$-2,6)(thf) (8-Dy). To a solution of **4-Dy** (183 mg, 0.207 mmol) in toluene (3 mL) a solution of 9-BBN (50.6 mg, 0.207 mmol) in THF (3 mL) was added and

stirred for 3 h at ambient temperature. Yellow, single crystals suitable for XRD analysis formed from a toluene solution (36.7 mg, 0.044 mmol, 21%). Elemental analysis (%) calculated for $BN_7C_{40}H_{65}DyO$ (833.32 g/mol): C 57.65, H 7.86, N 11.77; found: C 57.39, H 7.67, N 12.14.

$Tp^{tBu,Me}Dy(\mu_2-CH_2)(\mu_2-HBC_8H_{14})(HNC_6H_3iPr_2-2,6)$ (9-Dy). To a solution of **3-Dy** (150 mg, 0.170 mmol) in toluene (10 mL) a solution of 9-BBN (124 mg, 0.510 mmol) in toluene (5 mL) was added. The reaction mixture was stirred for 1 h at 40° C. Single crystals suitable for XRD analysis formed from a toluene solution.

$Tp^{tBu,Me}Dy(CO_2)_2(NC_6H_3iPr_2-2,6)$ (10-Dy). A solution of **4-Dy** (82.2 mg, 0.0931 mmol) in toluene (2 mL) under 1 bar CO_2 pressure was stirred for 30 minutes. After the color changed from yellow to colorless the solvent was removed under vacuum and the powder was washed with *n*-hexane (3x 5 mL) (44.6 mg, 0.0527 mmol, 57%). Elemental analysis (%) calculated for $BN_7C_{38}H_{57}DyO_4$ (849.23 g/mol): C 53.74, H 6.77, N 11.55; found: C 54.63 H 6.92, N 11.54.

Crystallography

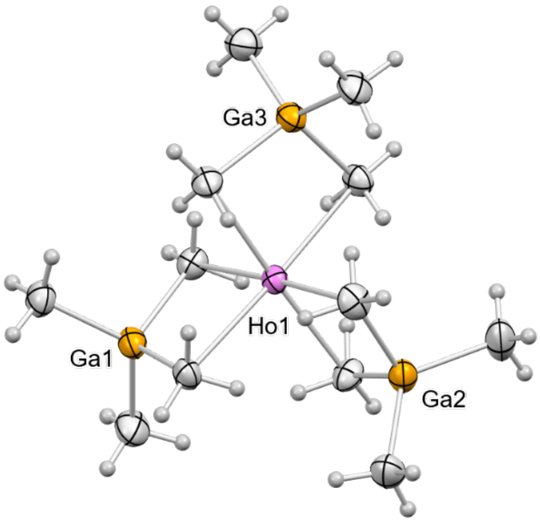

Figure S1. Crystal structure of $Ho(Ga(CH_3)_4)_3$. Atomic displacement ellipsoids were set at 50% probability. Selected interatomic distances [Å] and angles [°]: Ho1–C1 2.508(5), Ho1–C6 2.519(5), Ho1–C5 2.516(5), Ho1–C2 2.521(5), Ho1–C10 2.523(5), Ho1–C9 2.534(5), Ga1–C3 1.978(6), Ga1–C4 1.981(5), Ga1–C2 2.108(5), Ga1–C1 2.108(5); C1–Ho1–C6 90.60(17), C1–Ho1–C5 90.64(17), C1–Ho1–C2 86.84(16), C6–Ho1–C2 91.98(16), C1–Ho1–C10 177.28(17).

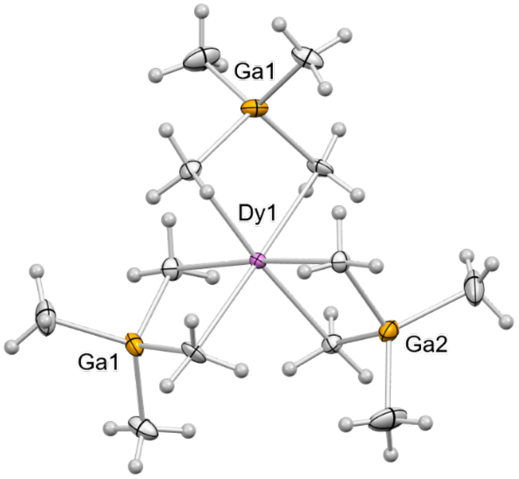

Figure S2. Crystal structure of $Dy(Ga(CH_3)_4)_3$. Atomic displacement ellipsoids were set at 50% probability. Selected interatomic distances [Å] and angles [°]:Ga1–C1 2.113(3), Dy1–C1 2.526(2), Ga1–C2 2.109(3), Dy1–C2 2.521(2), Ga1–C3 1.973(3), Dy1–C5 2.511(2), Ga2–C5 2.118(2), Dy1–C5' 2.511(2), Dy1–C2' 2.521(2), Dy1–C1' 2.527(2); C5–Dy1–C5' 87.06(11), C5–Dy1–C2 91.56(8), C5'–Dy1–C2 89.19(8), C2–Dy1–C2' 178.97(12), C5–Dy1–C1 91.53(9), C5–Dy1–C1' 175.30(8), C2–Dy1–C1 86.36(8), C2'–Dy1–C1 92.91(9), C5–Dy1–C1' 175.30(8), C5'–Dy1–C1' 91.54(9), C2–Dy1–C1' 92.91(9), C2'–Dy1–C1' 86.36(8), C1–Dy1–C1' 90.21(12).

' -x+1, y, -z+1/2

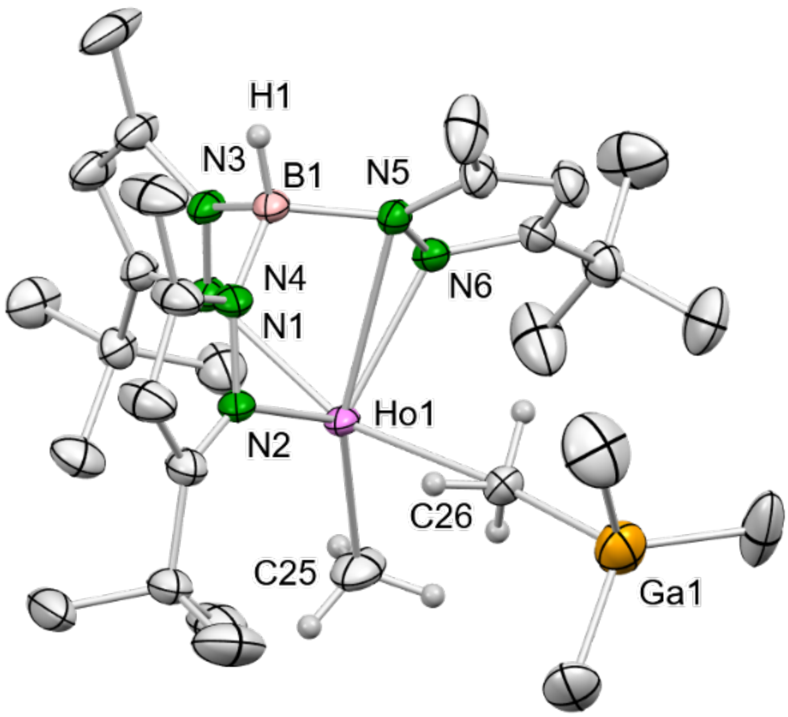

Figure S3. Crystal structure of **1-Ho**$^{\mathbf{Ga}}$. Atomic displacement ellipsoids were set at 50% probability. Hydrogen atoms except for the B–H and H–C25/26, and the disorder of one *tert*-butyl group are omitted for clarity. Selected interatomic distances [Å] and angles [°]: Ho1–N2 2.362(3), Ho1–N4 2.376(3), Ho1–N6 2.334(3), Ho1–C25 2.356(5), Ho1–C26 2.652(4); N2–Ho1–N4 77.82(12), N2– Ho1–N6 102.08(12), N2– Ho1–C25 128.40(19), N2– Ho1–C26 87.39(13), N4– Ho1–N6 75.50(12), N4–Ho1–C25 113.76(17), N4–Ho1–C26 152.60(12), N6–Ho1–C25 129.46(19), N6–Ho1–C26 85.41(13), C25–Ho1–C26 93.53(17), Ho1–C26–Ga1 174.9(2).

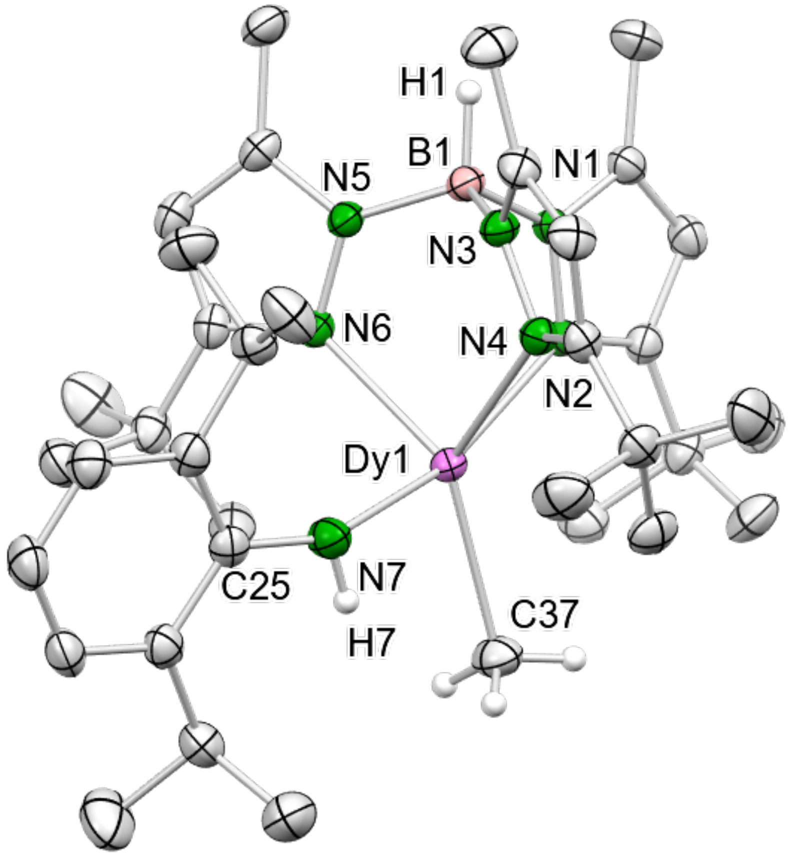

Figure S4. Crystal structure of **2-Dy**. Atomic displacement ellipsoids were set at 50% probability. Hydrogen atoms except for the B–H, the N–H and the H–C37, and the disorder of one *tert*-butyl group are omitted for clarity. Selected interatomic distances [Å] and angles [°]: Dy1–N2 2.5163(18), Dy1–N4 2.3752(17), Dy1–N6 2.4108(17), Dy1–N7 2.212(2), Dy1–C37 2.436(2); N2–Dy1–N4 74.52(6), N2–Dy1–N6 75.11(6), N2–Dy1–N7 166.20(7), N2–Dy1–C37 107.08(7), N4–Dy1–N6 94.33(6), N4–Dy1–N7 98.15(7), N4–Dy1–C37 130.41(8), N6–Dy1–N7 94.13(7), N6–Dy1–C37 134.76(8), N7–Dy1–C37 86.61(8), Dy1–N7–C25 155.84(16).

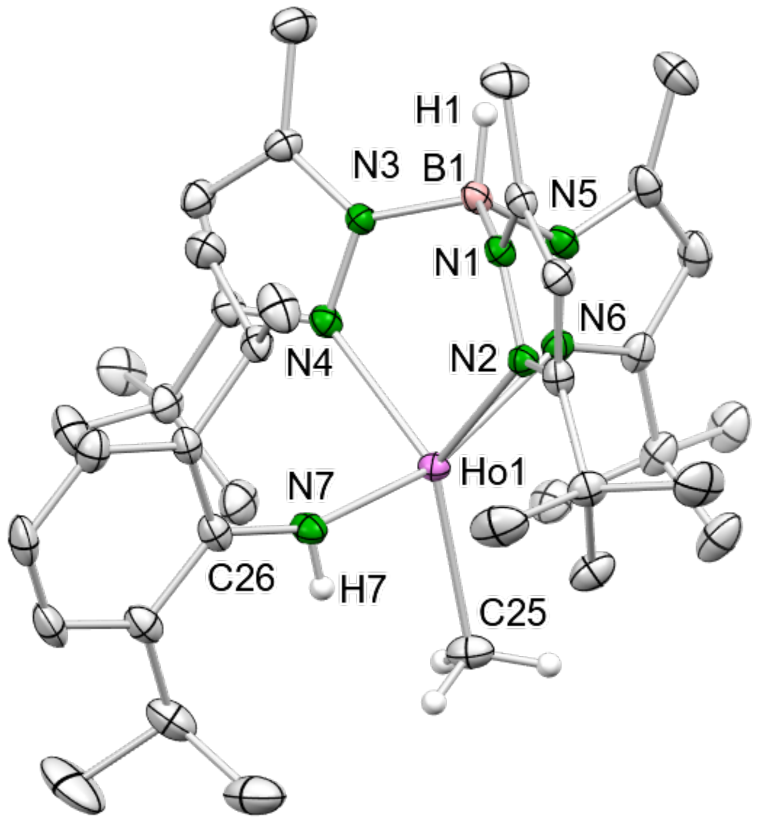

Figure S5. Crystal structure of **2-Ho**. Atomic displacement ellipsoids were set at 50% probability. Hydrogen atoms except for the B–H, the N–H and the H–C37, and one additional toluene molecule are omitted for clarity. Selected interatomic distances [Å] and angles [°]: Ho1–N2 2.373(2), Ho1–N4 2.369(2), Ho1–N6 2.536(2), Ho1–N7 2.222(2), Ho1–C25 2.427(3); N2–Ho1–N4 94.66(8), N2–Ho1–N6 77.23(7), N2–Ho1–N7 94.00(8), N2–Ho1–C25 129.46(9), N4–Ho1–N6 73.22(7), N4–Ho1–N7 94.60(8), N4–Ho1–C25 135.29(9), N6–Ho1–N7 164.08(8), N6–Ho1–C25 106.18(9), N7–Ho1–C25 89.66(9), Ho1–N7–C26 160.28(19).

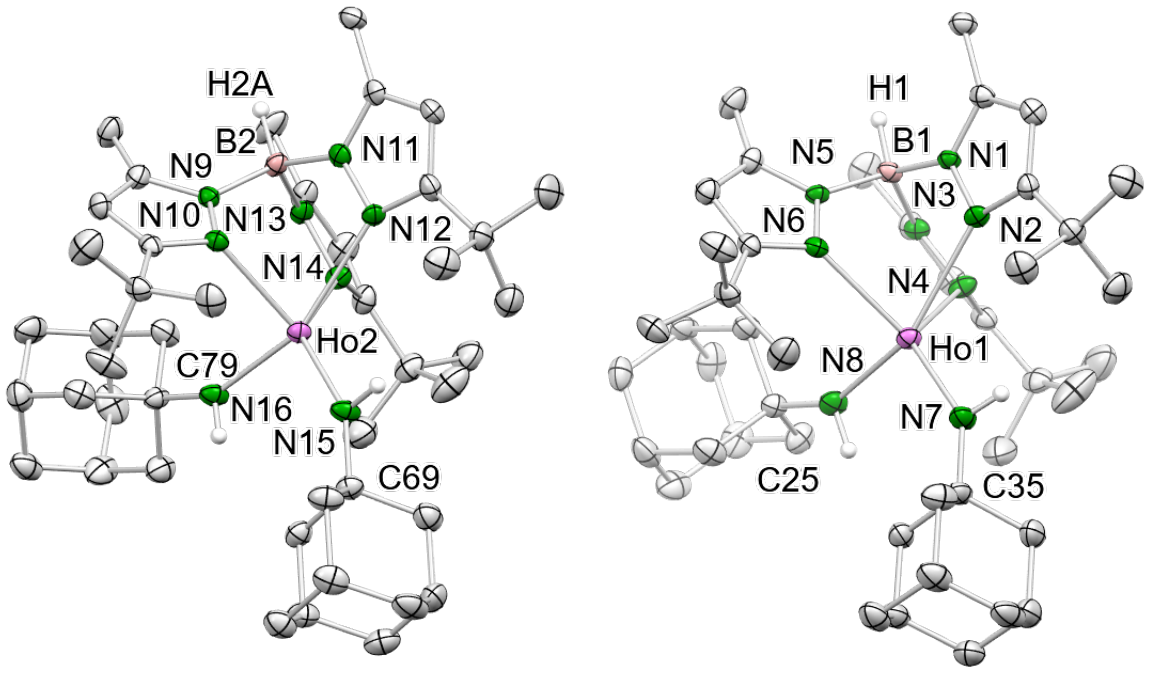

Figure S6. Crystal structure of **3-Ho**. Atomic displacement ellipsoids were set at 50% probability. Hydrogen atoms except for the B–H, the N–H, and lattice solvent (two molecules of THF) are omitted for clarity. The unit cell contains two independent molecules with different parameters. Selected interatomic distances [Å] and angles [°]: Ho1–N2 2.610(3), Ho1–N4 2.406(3), Ho1–N6 2.417(3), Ho1–N7 2.172(3), Ho1–N8 2.170(3), Ho2–N10 2.393(3), Ho2–N12 2.586(3), Ho2–N14 2.410(3), Ho2–N15 2.163(3), Ho2–N16 2.167(3); N2–Ho1–N4 69.77(10), N2–Ho1–N6 72.87(10), N2–Ho1–N7 103.30(12), N2–Ho1–N8 152.73(11), N4–Ho1–N6 100.02(10), N4–Ho1–N7 124.13(13), N4–Ho1–N8 91.54(12), N6–Ho1–N7 131.97(13), N6–Ho1–N8 91.94(12), N7–Ho1–N8 103.66(13), Ho1–N7–C35 144.0(3), Ho1–N8–C25 148.5(3), N10–Ho2–N12 71.81(10), N10–Ho2–N14 101.06(10), N10–Ho2–N15 119.74(12), N10–Ho2–N16 94.22(12), N12–Ho2–N14 70.46(11), N12–Ho2–N15 102.99(12), N12–Ho2–N16 154.81(11), N14–Ho2–N15 134.82(12), N14–Ho2–N16 92.71(12), N15–Ho2–N16 102.15(13), Ho2–N15–C69 146.9(3), Ho2–N16–C79 148.1(3).

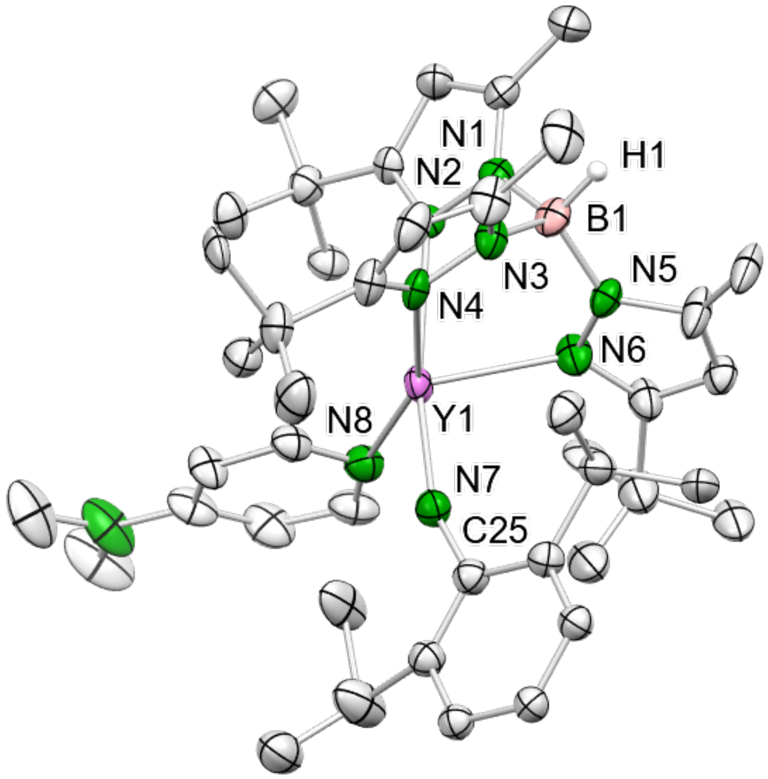

Figure S7. Crystal structure of **4-Y**. Atomic displacement ellipsoids were set at 50% probability. Hydrogen atoms except for the B–H, and three additional THF molecules as well as the disorder in two *tert*-butyl groups, one methyl group, and in the aryl group are omitted for clarity. Selected interatomic distances [Å] and angles [°]: Y1–N2 2.561(13), Y1–N4 2.457(14), Y1–N6 2.425(14), Y1–N7 2.26(3), Y1–N8 2.347(14); N2–Y1–N4 73.4(4), N2–Y1–N6 72.9(5), N2–Y1–N7 161.7(7), N2–Y1–N8 104.1(5), N4–Y1–N6 96.1(5), N4–Y1–N7 92.3(9), N4–Y1–N8 158.7(5), N6–Y1–N7 99.7(9), N6–Y1–N8 103.4(5), N7–Y1–N8 93.9(8), Y1–N7–C25 158(2).

* only connectivity, bond lengths and angles are not reliable

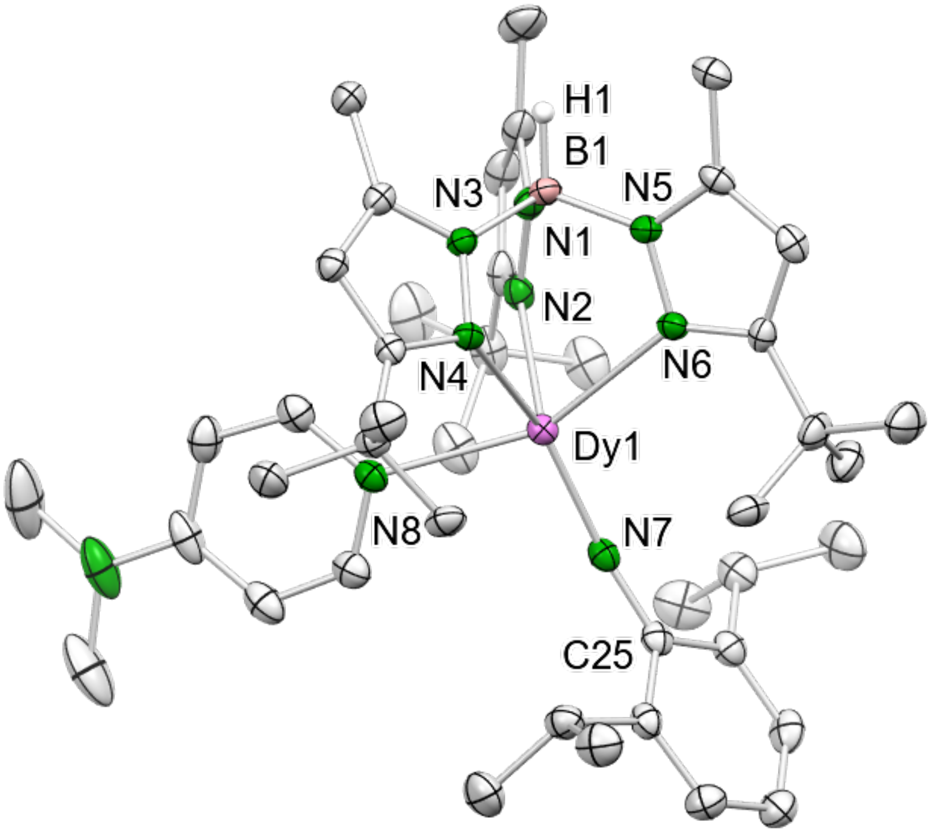

Figure S8. Crystal structure of **4-Dy**. Hydrogen atoms except for the B–H, and one additional toluene molecule are omitted for clarity. Atomic displacement ellipsoids were set at 50% probability. Selected interatomic distances [Å] and angles [°]: Dy1–N2 2.452(3), Dy1–N4 2.517(3), Dy1–N6 2.450(3), Dy1–N7 2.017(3), Dy1–N8 2.450(3); N2–Dy1–N4 92.37(9), N2–Dy1–N6 83.43(9), N2–Dy1–N7 120.04(10), N2–Dy1–N8 90.02(9), N4–Dy1–N6 73.88(9), N4–Dy1–N7 147.59(10), N4–Dy1–N8 80.27(9), N6–Dy1–N7 107.81(10), N6–Dy1–N8 152.99(9), N7–Dy1–N8 98.18(10), Dy1–N7–C25 166.0(2).

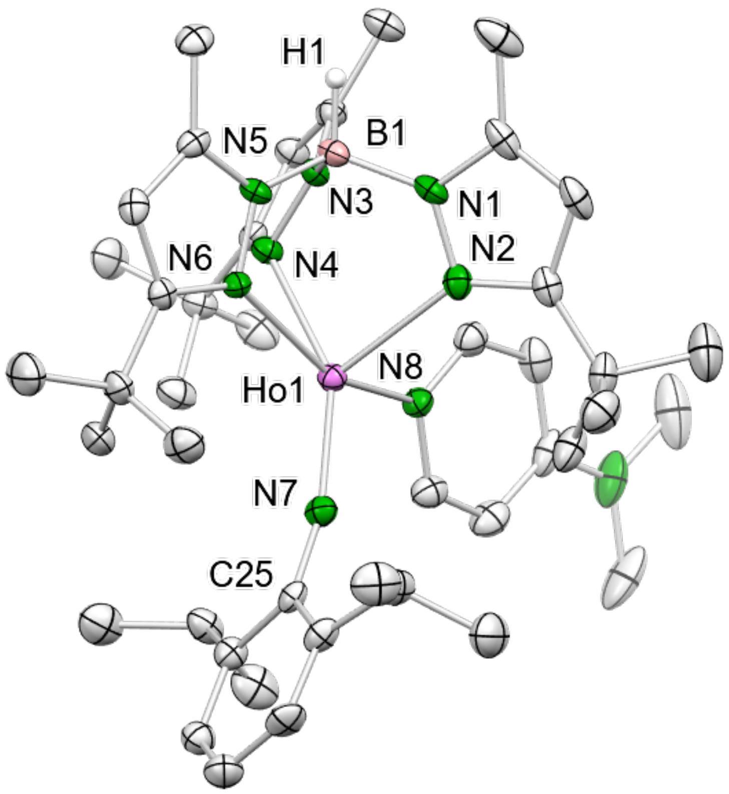

Figure S9. Crystal structure of **4-Ho**. Hydrogen atoms except for the B–H, and one additional toluene molecule are omitted for clarity. Atomic displacement ellipsoids were set at 50% probability. Selected interatomic distances [Å] and angles [°]: Ho1–N2 2.426(4), Ho1–N4 2.492(4), Ho1–N6 2.436(3), Ho1–N7 2.012(4), Ho1–N8 2.429(4); N2–Ho1–N4 93.34(12), N2–Ho1–N6 84.01(12), N2–Ho1–N7 120.38(13), N2–Ho1–N8 89.92(12), N4–Ho1–N6 74.40(12), N4–Ho1–N7 146.25(13), N4–Ho1–N8 79.83(12), N6–Ho1–N7 108.14(13), N6–Ho1–N8 153.09(12), N7–Ho1–N8 97.56(13), Ho1–N7–C25 166.7(3).

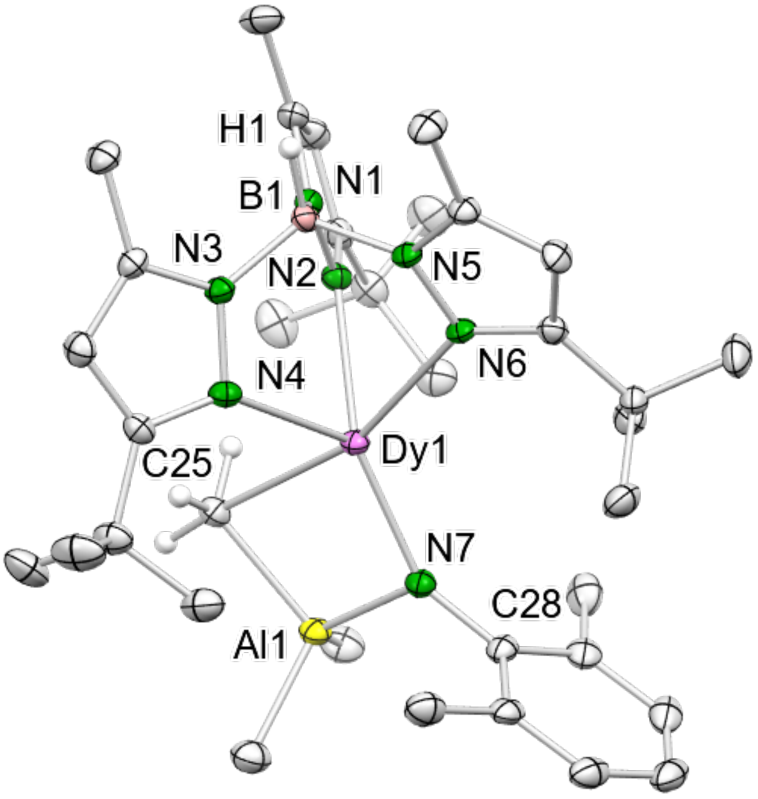

Figure S10. Crystal structure of **5-Dy**. Atomic displacement ellipsoids were set at 50% probability. Hydrogen atoms except for the B–H, and one additional toluene molecule are omitted for clarity. Selected interatomic distances [Å] and angles [°]: Dy1–N2 2.466(2), Dy1–N4 2.492(2), Dy1–N6 2.423(2), Dy1–N7 2.129(2), Dy1–C25 2.549(3), Al1–C25 2.091(3); N2–Dy1–N4 89.39(7), N2–Dy1–N6 74.80(7), N2–Dy1–N7 137.44(7), N2–Dy1–C25 90.18(8), N4–Dy1–N6 83.91(7), N4–Dy1–N7 131.58(7), N4–Dy1–C25 88.06(8), N6–Dy1–N7 114.82(8), N6–Dy1–C25 163.00(8), N7–Dy1–C25 81.61(8), Dy1–N7–C28 146.68(17).

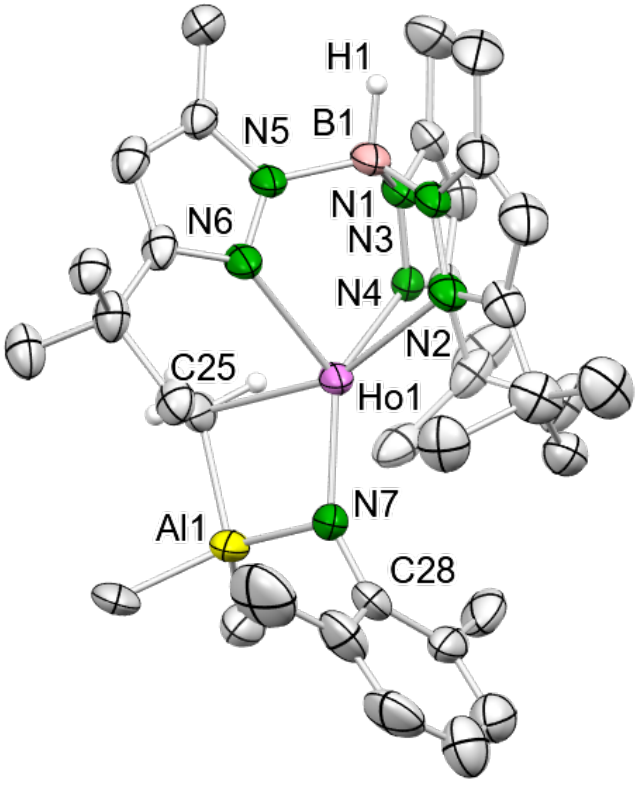

Figure S11. Crystal structure of **5-Ho**. Atomic displacement ellipsoids were set at 50% probability. Hydrogen atoms except for the B–H, the disorder of the *tert*-butyl groups and of the methyl groups of the trimethylaluminum are omitted for clarity. Selected interatomic distances [Å] and angles [°]: Ho1–N2 2.407(4), Ho1–N4 2.437(4), Ho1–N6 2.416(4), Ho1–N7 2.116(4), Ho1–C25 2.549(5), Al1–C25 2.105(6); N2–Ho1–N4 76.98(14), N2–Ho1–N6 77.74(15), N2–Ho1–N7 118.39(17), N2–Ho1–C25 159.64(16), N4–Ho1–N6 98.00(15), N4–Ho1–N7 132.41(16), N4–Ho1–C25 91.63(17), N6–Ho1–N7 128.39(16), N6–Ho1–C25 87.38(17), N7–Ho1–C25 81.76(17), Ho1–N7–C28 146.7(4).

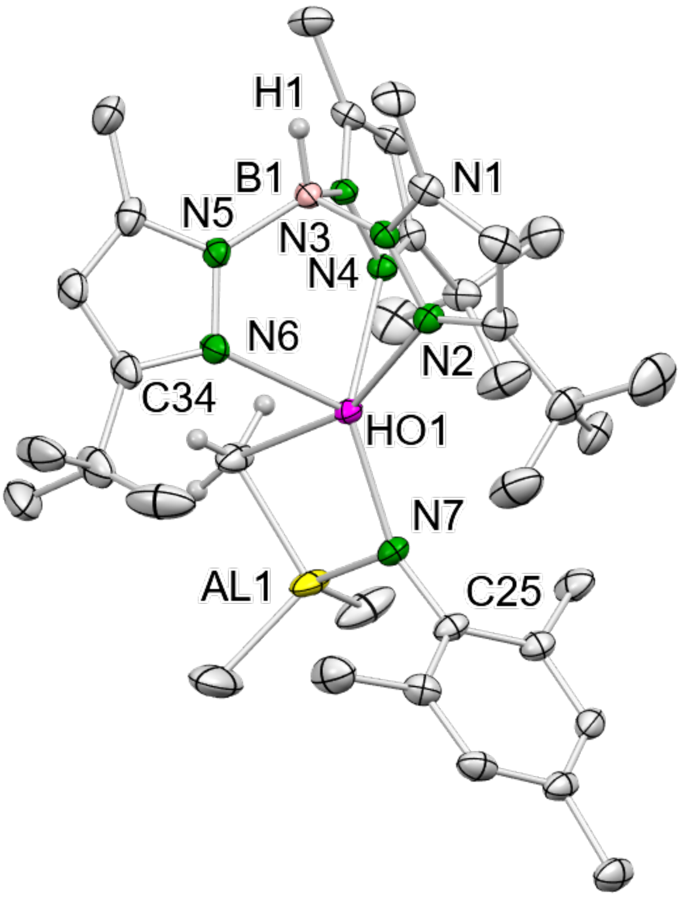

Figure S12. Crystal structure of **6-Ho**. Atomic displacement ellipsoids were set at 50% probability. Hydrogen atoms except for the B–H, and C34–H, one additional toluene molecule, and the disorder of the *tert*-butyl groups are omitted for clarity. Selected interatomic distances [Å] and angles [°]: Ho1–N2 2.394(3), Ho1–N4 2.436(3), Ho1–N6 2.454(3), Ho1–N7 2.116(3), Ho1–C34 2.554(3), Al1–C34 2.106(4); N2–Ho1–N4 76.67(9), N2–Ho1–N6 80.27(9), N2–Ho1–N7 116.96(10), N2–Ho1–C34 160.51(12), N4–Ho1–N6 95.45(9), N4–Ho1–N7 133.51(10), N4–Ho1–C34 89.54(11), N6–Ho1–N7 129.59(11), N6–Ho1–C34 87.52(13), N7–Ho1–C34 82.53(12), Ho1–N7–C25 151.0(2).

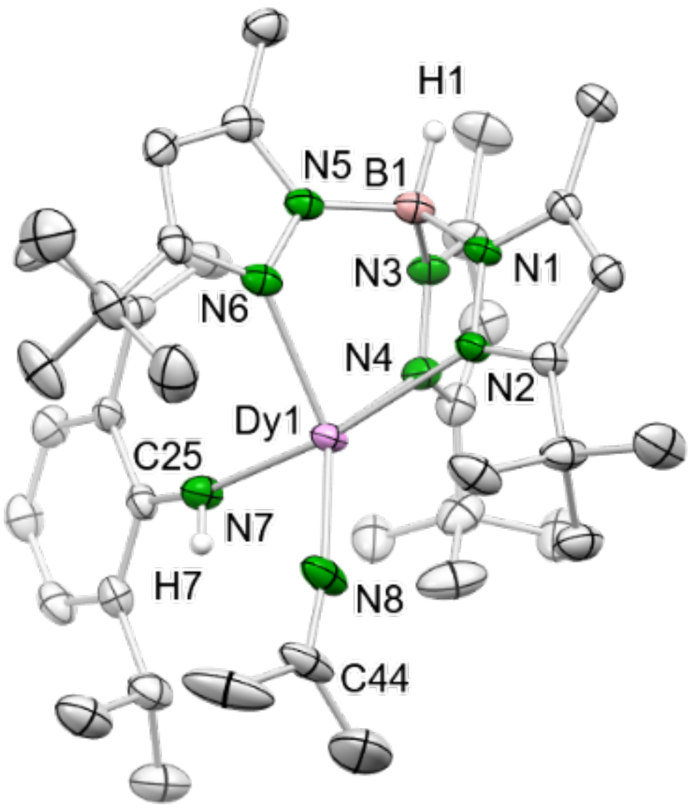

Figure S13. Crystal structure of **7-Dy**. Atomic displacement ellipsoids were set at 50% probability. Hydrogen atoms except for the B–H, and N–H are omitted for clarity. Selected interatomic distances [Å] and angles [°]: Dy1–N2 2.487(3), Dy1–N4 2.414(3), Dy1–N6 2.396(3), Dy1–N7 2.219(3), Dy1–N8 2.148(3); N2–Dy1–N4 75.05(10), N2–Dy1–N6 75.43(10), N2–Dy1–N7 167.88(11), N2–Dy1–N8 103.62(11), N4–Dy1–N6 95.88(10), N4–Dy1–N7 94.91(12), N4–Dy1–N8 136.15(12), N6–Dy1–N7 99.31(12), N6–Dy1–N8 126.77(12), N7–Dy1–N8 88.30(12), Dy1–N7–C25 156.3(3), Dy1–N8–C44 165.3(3).

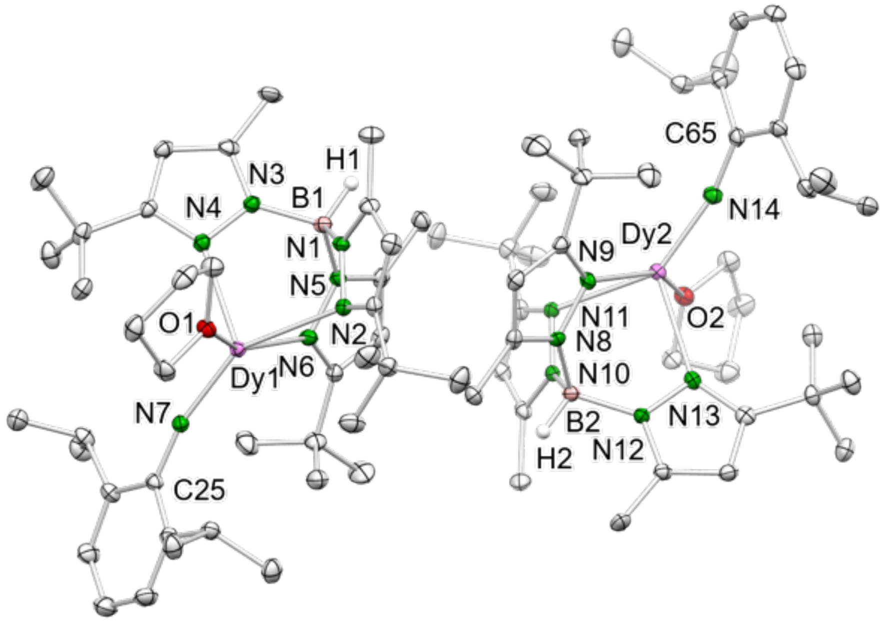

Figure S14. Crystal structure of **8-Dy**. Atomic displacement ellipsoids were set at 50% probability. Hydrogen atoms except for the B–H, and one additional THF molecule are omitted for clarity. The unit cell contains two independent molecules with different parameters. Selected interatomic distances [Å] and angles [°]: Dy1–N2 2.539(4), Dy1–N4 2.450(3), Dy1–N6 2.458(4), Dy1–N7 2.008(3), Dy1–O1 2.397(3), Dy2–N9 2.465(4), Dy2–N11 2.510(4), Dy2–N13 2.472(3), Dy2–N14 2.004(4), Dy2–O2 2.392(3); N2–Dy1–N4 92.82(12), N2–Dy1–N6 72.98(12), N2–Dy1–N7 149.01(13), N2–Dy1–O1 81.63(11), N4–Dy1–N6 82.59(12), N4–Dy1–N7 118.07(13), N4–Dy1–O1 91.16(11), N6–Dy1–N7 106.67(14), N6–Dy1–O1 153.45(11), N7–Dy1–O1 99.07(13), Dy1–N7–C25 166.9(3), N9–Dy2–N11 73.58(12), N9–Dy2–N13 83.99(11), N9–Dy2–N14 106.64(14), N9–Dy2–O2 151.86(11), N11–Dy2–N13 93.37(11), N11–Dy2–N14 144.82(14), N11–Dy2–O2 79.59(10), N13–Dy2–N14 121.80(14), N13–Dy2–O2 89.36(11), N14–Dy2–O2 100.11(13), Dy2–N14–C65 165.3(3).

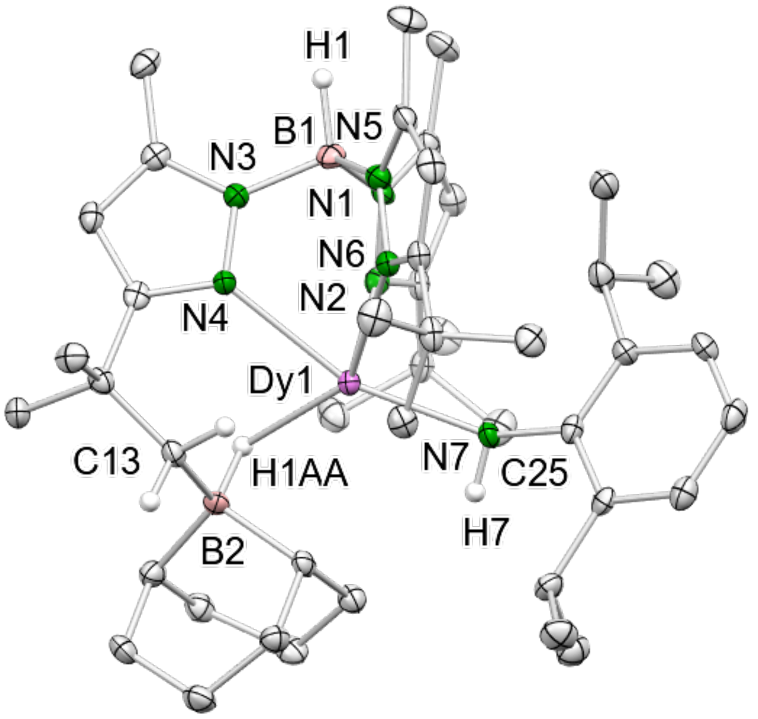

Figure S15. Solid-state structure of **9-Dy**. Atomic displacement ellipsoids were set at 50% probability. Hydrogen atoms except for the B–H, CH_2 and N–H are omitted for clarity. Selected interatomic distances [Å] and angles [°]: Dy1–N2 2.452(3), Dy1–N4 2.405(3), Dy1–N6 2.461(3), Dy1–N7 2.237(3), Dy1–H1AA 2.17(3), B2–C13 1.658(5); N2–Dy1–N4 79.55(9), N2–Dy1–N6 90.63(9), N2–Dy1–N7 93.18(10), N2–Dy1–H1AA 90.3(8), N4–Dy1–N6 70.77(9), N4–Dy1–N7 161.07(10), N4–Dy1–H1AA 75.5(8), N6–Dy1–N7 92.04(10), N6–Dy1–H1AA 145.4(8), N7–Dy1–H1AA 122.4(8), Dy1–N7–C25 143.4(2), Dy1–H1AA–B2 101.98.

Table S1. Crystallographic data for compounds **1-Ho**, **2-Dy**, and **2-Ho**

	**1-Ho**	**2-Dy**	**2-Ho**
formula	$C_{29}H_{55}GaBN_6Ho\cdot 1.5\ C_7H_8$	$C_{37}H_{61}BN_7Dy$	$C_{37}H_{61}BN_7Ho\cdot C_7H_8$
CCDC	2312222	2312224	2312220
M_r [g mol^{-1}]	871.45	777.23	871.80
color	colorless	colorless	colorless
crystal dimensions [mm^3]	0.557 x 0.340 x 0.082	0.198 x 0.117 x 0.091	0.214 x 0.176 x 0.089
cryst syst	triclinic	monoclinic	triclinic
space group	$P\overline{1}$	$P2_1/c$	$P\overline{1}$
a [Å]	9.8291(10)	11.6084(10)	10.767(5)
b [Å]	13.4472(12)	18.3503(15)	12.621(6)
c [Å]	18.5931(17)	18.8307(15)	17.188(9)
α [°]	96.149(2)	90	77.029(13)
β [°]	100.699(2)	98.396(2)	81.681(16)
γ [°]	110.456(2)	90	88.061(9)
V [Å^3]	2222.7(4)	3968.3(6)	2252.1(19)
Z	2	4	2
T [K]	100(2)	100(2)	173(2)
ρ_{calcd} [g cm^{-3}]	1.302	1.301	1.286
μ [mm^{-1}]	2.404	1.916	1.793
F (000)	898	1612	908
θ range [°]	1.645/30.507	1.558/30.507	1.228/28.699
unique reflns	74018	107390	59343
observed reflns	13452	12124	11646
R_1/wR_2 (I>2σ)[b]	0.0469/0.1284	0.0279/0.0623	0.0344/0.0907
R_1/wR_2 (all data)[b]	0.0601/0.1370	0.0368/0.0667	0.0371/0.0925
GOF[a]	1.090	1.052	1.057

[a]GOF = $[\Sigma w(F_0^2 - F_c^2)^2 / (n_0 - n_p)]^{1/2}$. [b]$R_1 = \Sigma(||F_0| - |F_c||) / \Sigma|F_0|$, $F_0 > 4\sigma(F_0)$. [c]$wR_2 = \{\Sigma[w(F_0^2 - F_c^2)^2 / \Sigma[w(F_0^2)^2]\}^{1/2}$.

Table S2. Crystallographic data for compounds **4-Y**, **4-Dy**, and **4-Ho**

	**4-Y***	**4-Dy**	**4-Ho**
formula	$C_{43}H_{67}BN_9Y\cdot 3\ C_4H_8O$	$C_{43}H_{67}BN_9Dy\cdot 1/2\ C_7H_8$	$C_{43}H_{67}BN_9Ho\cdot 1/2\ C_7H_8$
CCDC	2312214	2312225	2312219
M_r [g mol^{-1}]	1026.08	929.43	931.86
color	colourless	light yellow	yellow
crystal dimensions [mm^3]	0.267 x 0.176 x 0.072	0.145 x 0.081 x 0.056	0.107 x 0.105 x 0.054
cryst syst	monoclinic	monoclinic	monoclinic
space group	*P2₁*	*C2/c*	*C2/c*
a [Å]	11.4785(19)	41.940(3)	41.814(2)
b [Å]	17.511(3)	11.5627(7)	11.5565(6)
c [Å]	14.619(2)	19.9969(12)	20.0524(10)
α [°]	90	90	90
β [°]	105.928(4)	102.8940(10)	102.683(2)
γ [°]	90	90	90
V [Å^3]	2825.5(8)	9452.7(10)	9453.4(8)
Z	2	8	8
T [K]	100(2)	100(2)	100(2)
ρ_{calcd} [g cm^{-3}]	1.206	1.306	1.309
μ [mm^{-1}]	1.081	1.622	1.715
F (000)	1104	3872	3880
θ range [°]	1.449/ 22.520	1.830/28.720	1.832/28.508
unique reflns	34654	75914	68042
observed reflns	7276	12234	11080
R_1/wR_2 (I>2σ)[b]		0.0385/0.0773	0.0519/0.0805
R_1/wR_2 (all data)[b]		0.0629/0.0870	0.1002/0.0939
GOF[a]	1.110	1.022	1.013

[a]GOF = $[\Sigma w(F_0^2 - F_c^2)^2 / (n_0 - n_p)]^{1/2}$. [b]$R_1 = \Sigma(||F_0| - |F_c||) / \Sigma|F_0|$, $F_0 > 4\sigma(F_0)$, $wR_2 = \{\Sigma[w(F_0^2 - F_c^2)^2 / \Sigma[w(F_0^2)^2]\}^{1/2}$.

* Connectivity only.

Table S3. Crystallographic data for compounds **5-Dy**, **5-Ho** and **6-Ho**

	**5-Dy**	**5-Ho**	**6-Ho**
formula	$C_{35}H_{58}B_2N_7DyAl_{0.86}/Ga_{0.14}$ ·1/2 C_7H_8	$C_{35}H_{58}BN_7HoAl$	$C_{36}H_{60}BN_7HoAl$· C_7H_8
CCDC	2312218	2312216	2312215
M_r [g mol^{-1}]	829.43	779.60	885.76
color	yellow	colorless	yellow
crystal dimensions [mm^3]	0.341 x 0.072 x 0.039	0.315 x 0.067 x 0.028	0.273 x 0.173 x 0.150
cryst syst	monoclinic	triclinic	monoclinic
space group	$P2_1/n$	$P\overline{1}$	Cc
a [Å]	9.5640(11)	11.8446(8)	21.5531(9)
b [Å]	23.328(3)	12.0811(8)	11.2886(5)
c [Å]	18.369(2)	15.8693(11)	19.1515(8)
α [°]	90	81.388(2)	90
β [°]	100.874(2)	80.802(2)	108.0490(10)
γ [°]	90	66.125(2)	90
V [Å^3]	4024.6(8)	2040.7(2)	4430.3(3)
Z	4	2	4
T [K]	173(2)	100(2)	100(2)
ρ_{calcd} [g cm^{-3}]	1.369	1.269	1.328
μ [mm^{-1}]	2.005	1.991	1.843
F (000)	1714	804	1840
θ range [°]	2.079/30.550	1.852/24.850	1.988/28.770
unique reflns	73807	43797	35460
observed reflns	12298	7013	11130
R_1/wR_2 (I>2σ)[b]	0.0334/0.0719	0.0432/0.0908	0.0216/0.0429
R_1/wR_2 (all data)[b]	0.0462/0.0773	0.0656/0.0992	0.0237/0.0438
GOF[a]	1.042	1.046	1.015

[a]GOF = $[\Sigma w(F_0^2 - F_c^2)^2 / (n_0 - n_p)]^{1/2}$. [b]$R_1 = \Sigma(||F_0| - |F_c||) / \Sigma|F_0|$, $F_0 > 4\sigma(F_0)$, $wR_2 = \{\Sigma[w(F_0^2 - F_c^2)^2 / \Sigma[w(F_0^2)^2]\}^{1/2}$.

Table S4. Crystallographic data for compounds **7-Dy**, **9-Dy** and **3-Ho**

	**7-Dy**	**9-Dy**	**3-Ho**
formula	$C_{42}H_{64}BN_8Dy{\cdot}3/2\ C_7H_8$	$C_{44}H_{72}B_2N_7Dy$	$C_{44}H_{72}BN_8Ho{\cdot}\ C_4H_8O$
CCDC	2312217	2312221	2312223
M_r [g mol^{-1}]	956.49	883.20	960.94
color	yellow	colourless	colorless
crystal dimensions [mm^3]	0.121 x 0.090 x 0.080	0.141 x 0.141 x 0.064	0.184 x 0.128 x 0.064
cryst syst	monoclinic	monoclinic	triclinic
space group	$P2_1/n$	$C2/c$	$P\bar{1}$
a [Å]	12.1758(12)	42.723(6)	10.851(2)
b [Å]	17.8931(17)	10.3005(13)	20.269(4)
c [Å]	23.260(2)	21.324(2)	22.192(5)
α [°]	90	90	96.949(3)
β [°]	96.643(2)	90.449(2)	96.699(3)
γ [°]	90	90	100.067(3)
V [Å^3]	5033.5(8)	9384(2)	4722.7(17)
Z	4	8	4
T [K]	100(2)	100(2)	100(2)
ρ_{calcd} [g cm^{-3}]	1.262	1.250	1.351
μ [mm^{-1}]	1.524	1.628	1.719
F (000)	2000	3688	2016
θ range [°]	1.439/28.700	1.907/ 27.128	1.287/27.157
unique reflns	59881	49937	99190
observed reflns	12905	10376	20871
R_1/wR_2 ($I>2\sigma$)[b]	0.0439/0.0888	0.0376/0.0806	0.0412/0.0832
R_1/wR_2 (all data)[b]	0.0820/0.1048	0.0552/0.0888	0.0703/0.0941
GOF[a]	1.018	1.022	1.010

[a]GOF = $[\Sigma w(F_0^2 - F_c^2)^2 / (n_0 - n_p)]^{1/2}$. [b]$R_1 = \Sigma(||F_0| - |F_c||) / \Sigma|F_0|$, $F_0 > 4\sigma(F_0)$, $wR_2 = \{\Sigma[w(F_0^2 - F_c^2)^2 / \Sigma[w(F_0^2)^2]\}^{1/2}$.

Table S5. Crystallographic data for compounds **8-Dy**, **Ho[Ga(CH$_3$)$_4$]$_3$** and **Dy[Ga(CH$_3$)$_4$]$_3$**

	**8-Dy**	**Ho[Ga(CH$_3$)$_4$]$_3$**	**Dy[Ga(CH$_3$)$_4$]$_3$**
formula	$C_{40}H_{65}BN_7ODy\cdot 1/2\ C_4H_8O$	$C_{12}H_{36}Ga_3Ho$	$C_{12}H_{36}Ga_3Dy$
CCDC	2312226	2312212	2312213
M_r [g mol^{-1}]	869.35	554.50	552.07
color	yellow	pink	colorless
crystal dimensions [mm^3]	0.241 x 0.137 x 0.107	0.60 x 0.40 x 0.30	0.348 x 0.180 x 0.174
cryst syst	orthorhombic	tetragonal	monoclinic
space group	*Pna2$_1$*	*I4$_1$/a*	*C2/c*
a [Å]	19.7303(9)	17.6111(4)	10.8711(6)
b [Å]	11.5097(5)	17.6111(4)	15.7025(9)
c [Å]	37.3911(18)	26.4443(6)	12.4891(7)
α [°]	90	90	90
β [°]	90	90	102.0700(10)
γ [°]	90	90	90
V [Å^3]	8491.1(7)	8201.7(4)	2084.8(2)
Z	8	16	4
T [K]	100(2)	173(2)	100(2)
ρ_{calcd} [g cm^{-3}]	1.360	1.796	1.759
μ [mm^{-1}]	1.801	7.698	7.360
F (000)	3624	4288	1068
θ range [°]	1.089/30.487	4.834/ 29.234	3.078/ 29.144
unique reflns	219882	76662	16607
observed reflns	25876	5511	2800
R_1/wR_2 (I>2σ)[b]	0.0316/0.0645	0.0404/0.0773	0.0189/0.0479
R_1/wR_2 (all data)[b]	0.0371/0.0669	0.0434/0.0782	0.0196/0.0483
GOF[a]	1.040	1.395	1.065

[a]GOF = $[\Sigma w(F_0^2 - F_c^2)^2 / (n_0 - n_p)]^{1/2}$. [b]$R_1 = \Sigma(||F_0| - |F_c||) / \Sigma|F_0|$, $F_0 > 4\sigma(F_0)$, $wR_2 = \{\Sigma[w(F_0^2 - F_c^2)^2 / \Sigma[w(F_0^2)^2]\}^{1/2}$.

X-ray Crystallography and Crystal Structure Determinations. Single crystals of the reported compounds **1-Ln** – **9-Ln** were grown by standard techniques from saturated solutions using toluene. Suitable crystals for X-ray structure analyses were selected in a glovebox and coated with Parabar 10312 (previously known as Paratone N, Hampton Research) and fixed on a nylon loop/glass fiber.

X-ray data for above mentioned compounds, except for **1-Ho^{Ga}** and $Ho(Ga(CH_3)_4)_3$, were collected on a Bruker APEX II DUO (all remaining; instruments equipped with an IμS microfocus sealed tube and QUAZAR optics for MoKα (λ = 0.71073 Å) and CuKα (λ = 1.54184 Å) radiation. **1-Ho^{Ga}** was measured on a Bruker SMART APEX II instrument equipped with a fine focus sealed tube and TRIUMPH monochromator using MoK_α radiation (λ = 0.71073 Å). The data collection strategy was determined using COSMO[4] employing ω-scans. Raw data were processed using APEX II/III[5] and SAINT,[6] corrections for absorption effects were applied using SADABS.[7] Data for $Ho(Ga(CH_3)_4)_3$ were collected at 173(2) K on a STOE IPDS II system, equipped with a fine focus sealed tube and graphite monochromator using MoK_α radiation (λ = 0.71073 Å) performing ϕ-scans. Raw data were collected and integrated using Stoe's X-Area[8] software package. Absorption correction has been done using X-Red[9] and X-Shape[10]. The structures were solved by direct methods and refined against all data by full-matrix least-squares methods on F^2 using ShelXl[11] and ShelXle.[12] All graphics were produced employing ORTEP-3[13] and POV-Ray.[14] Further details of the refinement and crystallographic data are listed in Table S1-S5, and in the CIF files. CCDC depositions 2312212-2312226 contain all the supplementary crystallographic data for this paper. These data can be obtained free of charge from The Cambridge Crystallographic Data Centre via www.ccdc.cam.ac.uk/structures/.

DRIFT Spectroscopy

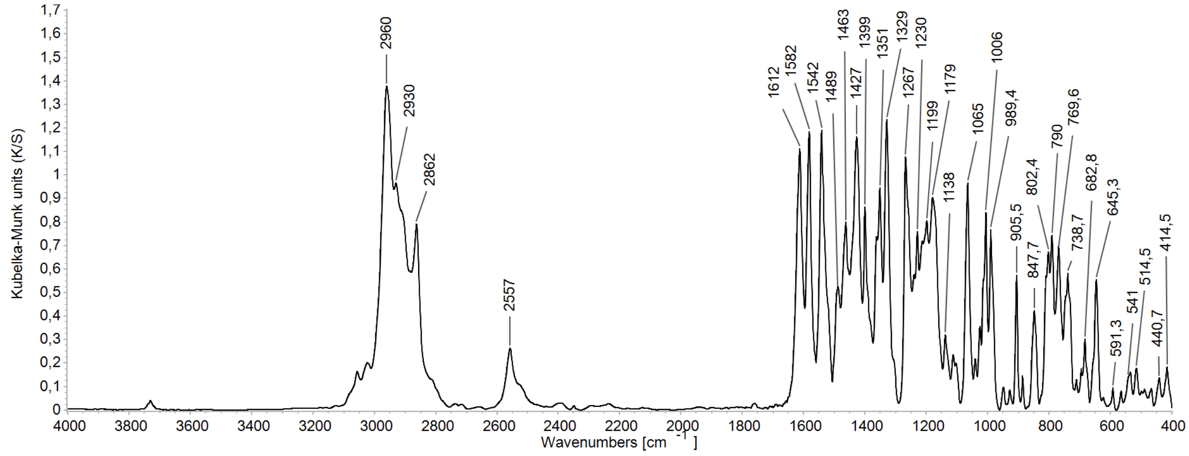

Figure S16. DRIFT spectrum (KBr) of compound **4-Dy** at 26 °C.

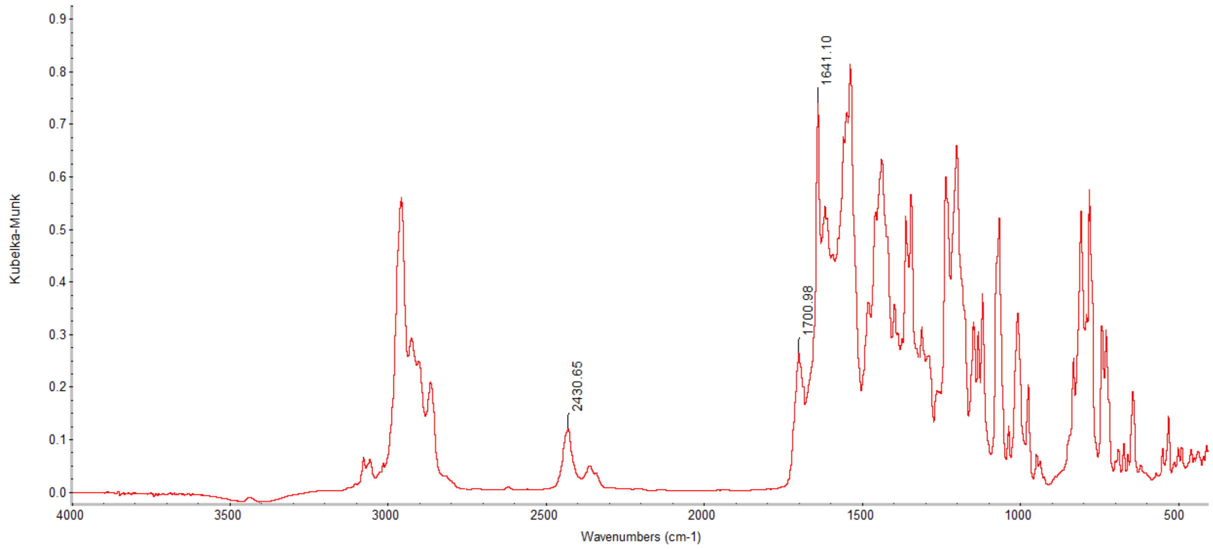

Figure S17. DRIFT spectrum (KBr) of compound **10-Dy** at 26 °C.

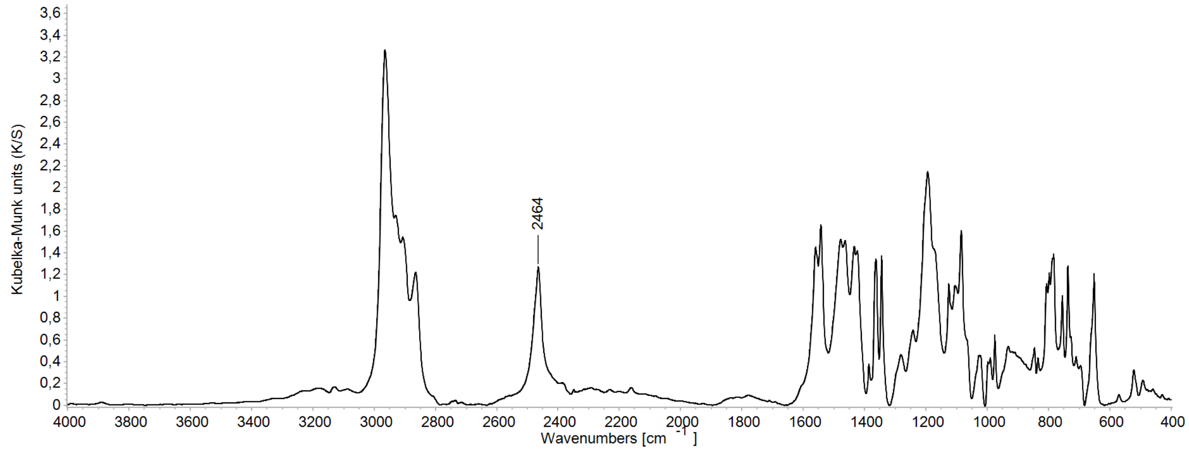

Figure S18. DRIFT spectrum (KBr) of compound of HTp^{tBu,Me} at 25 °C.

NMR spectroscopy

Notes on NMR spectroscopic characterizations.

Due to the paramagnetic nature of Dy(III) and Ho(III) any conclusive interpretation of the recorded NMR spectra was not possible. In general, solvent peaks are marked with an asterisk (*).

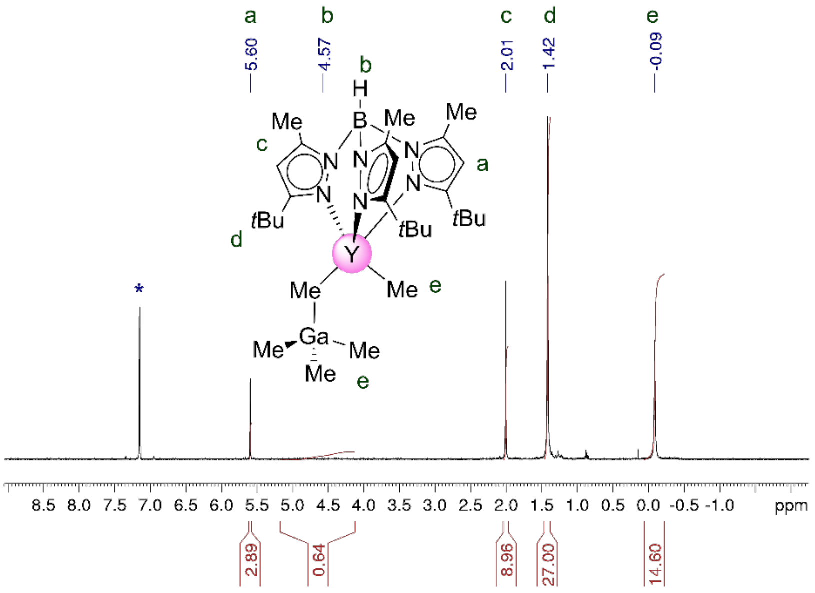

Figure S19. ^{1}H NMR spectrum (400 MHz) of compound **1-Y^{Ga}** in C_6D_6 at 26 °C.

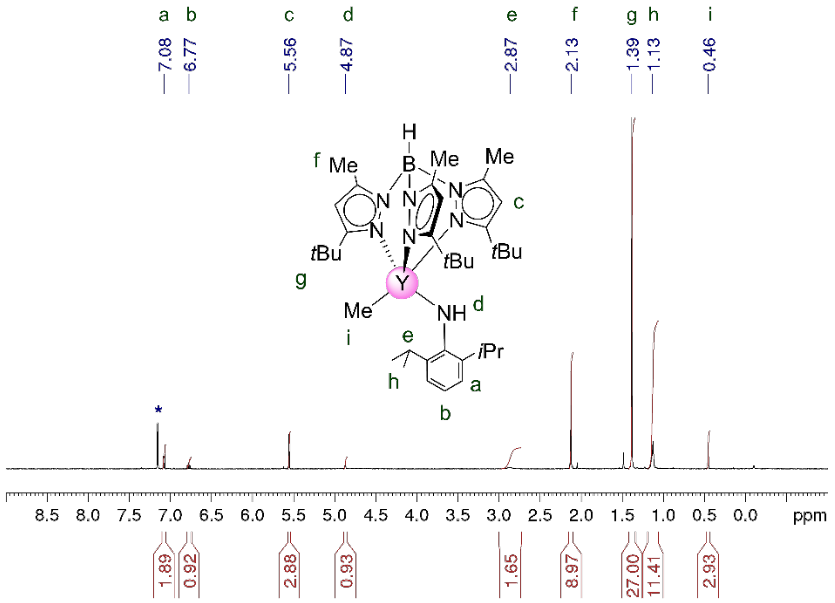

Figure S20. ^{1}H NMR spectrum (400 MHz) of compound **2-Y** in C_6D_6 at 26 °C.

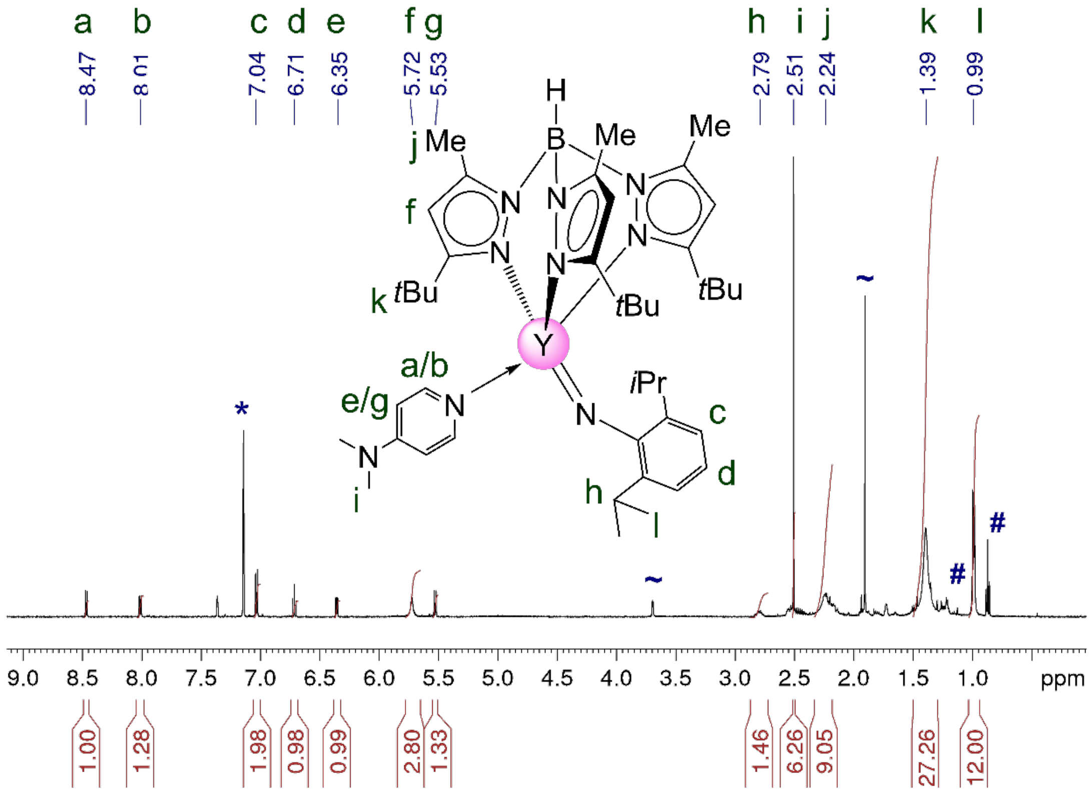

Figure S21. ^{1}H NMR spectrum (500 MHz) of compound **4-Y** in C_6D_6 at 26 °C.

References

[1] J. L. Kisko, T. Hascall, C. Kimblin and G. Parkin, *J. Chem. Soc., Dalton Trans.,* 1999, 1929-1936.

[2] M. Zimmermann, N. Å. Frøystein, A. Fischbach,. P. Sirsch, H. M. Dietrich, K. W. Törnroos, E. Herdtweck and R. Anwander, *Chem. Eur. J.*, 2007, **13**, 8784-8800.

[3] W. J. Evans, R. Anwander, R. J. Doedens and J. W. Ziller, *Angew. Chem. Int. Ed. Engl.*, 1994, **33**, 1641-1644.

[4] COSMO, v. 1.61; Bruker AXS Inc.: Madison, WI, 2012.

[5] APEX 3 V. 2019.11-0, Bruker AXS Inc., Madison, WI, 2019.
APEX *2 V. 2012.10_0*, Bruker AXS Inc., Madison, WI, 2012.

[6] *SAINT V. 8.38A*, Bruker AXS Inc., Madison, WI, 2017.
SAINT V. 8.40B, Bruker Nano, Inc., Madison, WI, 2019.

[7] SADABS: L. Krause, R. Herbst-Irmer, G. M. Sheldrick and D. Stalke, *J. Appl. Crystallogr.* 2015, **48**, 3.

[8] *X-Area v. 1.55*, Stoe & Cie GmbH, Darmstadt, Germany, 2009.

[9] *X-Red 32 v. 1.53*, Stoe & Cie GmbH, Darmstad, Germany, 2009.

[10] *X-Shape v.2.12.2*, Stoe & Cie GmbH, Darmstadt, Germany, 2009.

[11] ShelXL: Sheldrick, G. M., Crystal structure refinement with SHELXL, *Acta Cryst.* **2015***,* C71, 3-8

[12] ShelXle: C. B. Hübschle, G. M. Sheldrick and B. Dittrich, *J. Appl. Crystallogr.* 2011, **44**, 1281-1284.

[13] L. J. Farrugia, *J. Appl. Crystallogr.* 1997, **30**, 565-566.

[14] POV-Ray v. 3.6; Persistence of Vision Pty. Ltd.: Williamstown, Victoria, Australia, 2004. http://www.povray.org/ .
